# Toxicokinetics of the Antidepressant Fluoxetine and Its Active Metabolite Norfluoxetine in *Caenorhabditis elegans* and Their Comparative Potency

**DOI:** 10.1021/acs.est.3c07744

**Published:** 2024-02-12

**Authors:** Merel A. van der Most, Wouter Bakker, Sebastiaan Wesseling, Nico W. van den Brink

**Affiliations:** Division of Toxicology, Wageningen University and Research, Wageningen 6708 WE, The Netherlands

**Keywords:** toxicokinetics, Caenorhabditis elegans, fluoxetine, norfluoxetine, behavior, bioaccumulation

## Abstract

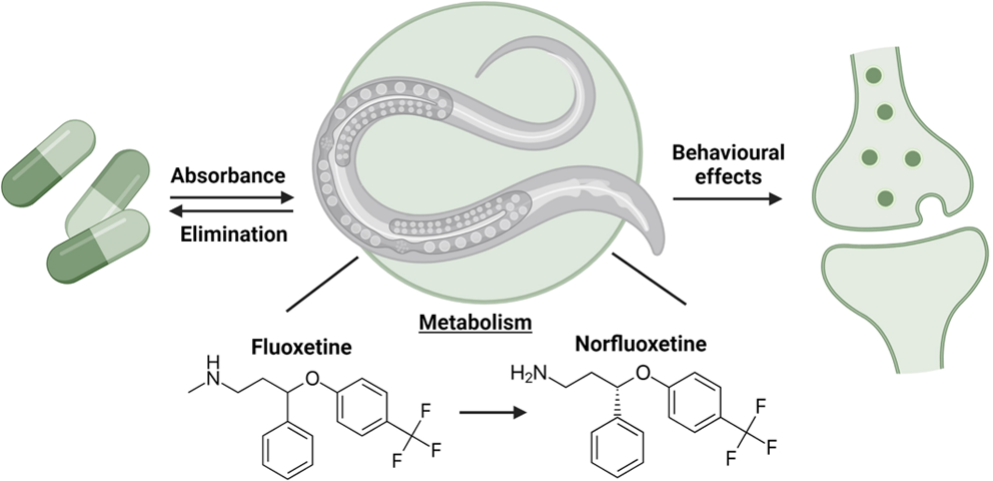

The nematode *Caenorhabditis elegans* is a valuable model for ecotoxicological research, yet limited attention has been given to understanding how it absorbs, distributes, metabolizes, and excretes chemicals. This is crucial for *C. elegans* because the organism is known to have strong uptake barriers that are known to be susceptible to potential confounding effects of the presence of *Escherichia coli* as a food source. One frequently studied compound in *C. elegans* is the antidepressant fluoxetine, which has an active metabolite norfluoxetine. In this study, we evaluated the toxicokinetics and relative potency of norfluoxetine and fluoxetine in chemotaxis and activity tests. Toxicokinetics experiments were conducted with varying times, concentrations of fluoxetine, and in the absence or presence of *E. coli*, simulated with a one-compartment model. Our findings demonstrate that *C. elegans* can take up fluoxetine and convert it into norfluoxetine. Norfluoxetine proved slightly more potent and had a longer elimination half-life. The bioconcentration factor, uptake, and elimination rate constants depended on exposure levels, duration, and the presence of *E. coli* in the exposure medium. These findings expand our understanding of toxicokinetic modeling in *C. elegans* for different exposure scenarios, underlining the importance of considering norfluoxetine formation in exposure and bioactivity assessments of fluoxetine.

## Abstract 2

This study explores the toxicokinetic mechanisms in *C. elegans*, a valuable model for the replacement, refinement, and reduction of animal experiments, focusing on the antidepressant fluoxetine under different exposure scenarios.

## 1 Introduction

*Caenorhabditis elegans* is a model organism that has become increasingly popular as a model for ecotoxicological studies.^1,2^ This nematode allows for the study of whole organism responses to chemicals, while it has a relatively short lifecycle and ease of handling while still being considered an *in vitro* technique.^1,2^*C. elegans* is a good model for neuroactive compounds because of the well-conserved signaling pathways, well-characterized behavioral responses, and the existence of mutants and molecular biomarkers through which mechanistic information on the mode of toxicity can be obtained.^1−4^ An example of a neuroactive compound that has been frequently studied in *C. elegans* is fluoxetine, an antidepressant and, more specifically, a selective serotonin reuptake inhibitor (SSRI). It inhibits the serotonin reuptake transporter and therefore causes serotonin to remain in the synaptic cleft for longer periods of time.^5,6^ Effects of fluoxetine have been observed at low, environmentally relevant concentrations for a variety of species, such as the freshwater shrimp *Gammarus Pulex*(7) and the fathead minnow *Pimephales promelas*.^8^*C. elegans* has been frequently used for studies on the mechanism of action, molecular targets, and behavioral and metabolic effects of fluoxetine,^5,9−14^ but data on the toxicokinetics of fluoxetine and its major metabolite norfluoxetine in *C. elegans* are lacking.

Toxicokinetics refers to the study of the absorption, distribution, metabolism, and excretion (ADME) of chemicals. Overall, the number of toxicokinetic experiments in *C. elegans* is still limited^15−20^ and there are only a handful of studies on *in silico* models that describe these toxicokinetic processes in *C. elegans*.^21−25^ To have a proper insight into the toxicity of a chemical, a clear understanding of the toxicokinetics is important. *C. elegans* has two different pathways of chemical uptake: across the cuticle or via ingestion.^21−24^ Absorption through the cuticle can happen through passive diffusion or active transport, but it is known to be a strong barrier to uptake, which is often mentioned as one of the downsides of using *C. elegans* as a model organism for other environmental species.^21,26^ Uptake via ingestion occurs through pharyngeal pumping, where particles (including *Escherichia coli*) and liquids are taken up.^21,23^ However, the pharynx also acts as a barrier to xenobiotics because most of the liquid is expelled. Chemicals bound to bacteria will still be taken up in the intestine.^21,23^ The presence of *E. coli* in the exposure medium can thus play an important role in the toxicokinetics of *C. elegans* not only through increasing uptake via ingestion and stimulation of pharyngeal pumping but also because they affect lipid levels in the nematodes and thus the associated potential for the storage of xenobiotics in these lipids.^21,23^ Effects of bacteria on uptake kinetics have been shown for, for example, phenanthrene^23^ and iron nanoparticles,^24^ but these studies were only performed with one exposure concentration and metabolism was not included. *C. elegans* does not have a liver but expresses different phase I enzymes, such as 86 cytochrome P450s genes, in its somatic cells, so it is also able to metabolize compounds.^21,22,26^ However, these kinetics of metabolism have only been tested for chlorpyrifos.^22^

Fluoxetine also has an active metabolite, norfluoxetine, which has been quantified in the environment at concentrations similar to fluoxetine.^27^ The toxicokinetics of both fluoxetine and norfluoxetine are therefore important to assess when considering the potential risks of fluoxetine exposures. Furthermore, previous studies into the effects of fluoxetine on *C. elegans* locomotion behavior found that nematode activity stabilized or even recovered over time under continued exposure,^14^ which raises the question on how fast fluoxetine is taken up and detoxified or excreted by *C. elegans* and what the contribution is of its allegedly active metabolite norfluoxetine. The current study therefore aims to gain more information about the toxicokinetics of fluoxetine and its major metabolite norfluoxetine in *C. elegans*, in the presence and absence of *E. coli*, while also considering the influence of concentration and exposure time. The relative toxicity of norfluoxetine compared to fluoxetine was also tested for behavioral end points including activity and chemotaxis.

## 2 Materials and Methods

### 2.1 Materials and Test Species Maintenance

*C. elegans* Bristol N2 strain and *E. coli* OP50 and NA22 strains were obtained from the Caenorhabditis Genetics Center (CGC, University of Minnesota, Minneapolis). Fluoxetine hydrochloride (FLX) (racemic mixture, 100%) was obtained from Merck (Zwijndrecht, Netherlands), (*R*)-norfluoxetine hydrochloride (>98%) and (*S*)-norfluoxetine hydrochloride (>98%) from Aobious (Gloucester), fluoxetine-D5 (>99%) from Biosynth S.R.O. (Bratislava, Slovakia), and 5-fluoro-2′-deoxyuridine from Merck (Zwijndrecht, Netherlands). *C. elegans* was maintained on a nematode growth medium (NGM) with OP50 according to the protocol reported by Stiernagle.^28^ Approximately 1 week before each experiment, *C. elegans* was transferred to peptone-enriched plates seeded with NA22, which allows for the development of a large culture.^29^*C. elegans* cultures from a single plate were age-synchronized through bleaching with a mixture of sodium hypochlorite, sodium hydroxide, and MilliQ water and then left to hatch overnight in M9 buffer.^28^ Age synchronization was confirmed by visual inspection under a stereomicroscope. L1 larvae were then transferred to 24-well plates with *S* medium^28^ and *E. coli* OP50 at an optical density at 600 nm (OD600) of 0.65–0.70 and let to develop until exposure.

### 2.2 Behavioral End Points

Effects of fluoxetine and norfluoxetine (racemic mixture) on *C. elegans* chemotaxis and activity behavior were tested. *C. elegans* was exposed in *S* medium with *E. coli* at an OD600 of 0.4 to nine concentrations of fluoxetine and norfluoxetine, ranging from 1 ng/L to 100 mg/L. For the chemotaxis assay,^30^ after 72 h of exposure (starting at the L1 larval stage), 10 μL of nematode suspension was placed in the center of a Petri dish filled with NGM. Two quadrants were spiked with 2 μL of an attractant (0.5% diacetyl), and two contained 2 μL water as a control.^30^ After 45 min, during which the worms could move freely, the dish was moved to 4 °C to immobilize the worms, and the number of worms in each quadrant was counted.

The general activity was measured with a WMicrotracker from Phylumtech. This system measured the collective movement in multiwell plates by detecting the number of interruptions of an infrared beam over time. The total number of interruptions over a 30 min period was calculated with the Wmicrotracker software. As *C. elegans* develop, their activity increases.^31^ To adjust for variations in the signal caused by the number of worms in each well, a baseline measurement of activity was taken after 50 h (the point at which a stable activity was reached).^14^ After this, worms were exposed and exposure-related changes in activity were quantified continuously for 24 h for each well specifically. Further details on the chemotaxis and activity assays can be found in SI A1.

### 2.3 Toxicokinetic Experiments

#### 2.3.1 Fluoxetine Concentrations and Stability in Medium and Bacteria

To check the stability of fluoxetine in the medium over time, 450 μL of *S* medium was spiked with 50 μL of fluoxetine to a final concentration of 10 mg/L, similar to exposure conditions. After 0.5, 1, 2, 5, 10, and 24 h at 20 °C, samples of spiked medium were stored at −80 °C until analysis. Uptake and metabolism in *E. coli* OP50 over time were checked by adding *E. coli* to 450 μL of *S* medium at an OD600 of 1.0 in a 24-well plate and adding 50 μL of fluoxetine at a final concentration of 10 mg/L. After 0.5, 1, 2, 5, 10, and 24 h at 20 °C, samples were centrifuged for 5 min at 15,000*g*. Fluoxetine is not only taken up by *E. coli* but it can also externally adsorb to it. Washing will remove the externally adsorbed *E. coli*, which can underestimate bacteria-associated fluoxetine concentrations. Therefore, two replicates of the samples were washed three times with M9 buffer, while the other two were just centrifuged once, and the entire supernatant was removed. The supernatant (*S* medium with fluoxetine) was stored at −20 °C, 50 μL of the internal standard fluoxetine-D5 was added to the pellet at a concentration of 10 μM, and samples were stored at −80 °C until analysis.

#### 2.3.2 Uptake and Elimination of Fluoxetine and Norfluoxetine in *C. elegans*

Age synchronous L1 *C. elegans* was left to develop in *S* medium^28^ with *E. coli* (OD600 of 0.4) for 48 h and was then exposed at 20 °C in four different scenarios (Table 1). For the long-term experiment IV, the exposure of L4 larvae and adult worms was compared, and for the adult group, the worms were left to develop for an extra 24 h. To limit egg-laying and prevent egg-hatching in experiment IV, 25 μM of 5-fluoro-2′-deoxyuridine (FUDR) was added after 48 h.^32^

**Table 1:** Exposure Scenarios at 20 °C for Determining Toxicokinetics of Fluoxetine and Norfluoxetine (Racemic Mixture) in *C. elegans*

exp	goal	concentrations	time points	*E. coli*?
I	quantify elimination (rate constant) after 1 h exposure	0.5, 5, and 20 mg/L for 1 h, then transferred to clean medium	1, 3, 6, 24 h	no
II	short-term uptake (rate constant) of different concentrations	0.5, 5, and 20 mg/L	0.5, 1, and 2 h	no
III	quantify effect of *E. coli* on uptake (food vs dermal uptake)	10 mg/L	0.5, 2, 5, and 10 h	no and yes
IV	compare long-term exposure of L4 and adult worms	0.5 and 20 mg/L	2, 5, 10, 24, and 48 h	yes

For each time point, worms were washed three times with M9 buffer to remove the remaining *E. coli* and the final pellet was resuspended in 50 μL of fluoxetine-D5 at 10 μM in 100% methanol as an internal standard (IS) and then frozen at −80 °C until extraction. Three replicates of 1500 pooled *C. elegans* were used for each time point for each concentration. For experiments with *E. coli*, the OD600 was kept constant at 0.4.

### 2.4 Sample Preparation

To quantify fluoxetine and norfluoxetine in the medium, samples were centrifuged at 15,000*g* for 15 min to spin down bacteria and/or *C. elegans* and then supernatants were diluted 4–50× in methanol depending on the expected concentration and stored at −20 °C until the LC-MS/MS measurement. *E. coli* containing pellets were resuspended in 250 μL of methanol and frozen in liquid nitrogen and then left at room temperature to thaw, followed by 5 min of sonication in a sonication bath. The freeze–thawing and sonication were repeated twice more and after this, samples were centrifuged at 15,000*g* for 15 min. The supernatant was stored at −20 °C until analysis.

*C. elegans* containing pellets were resuspended in 500 μL of methanol, and 0.6 g of ZiO_2_ ceramic beads (1.4 mm) were added to the vial. The samples were homogenized three times for 20 s using a Minilys homogenizer (Bertin Technologies) at middle speed. In between cycles, the samples were kept on ice. After homogenization, the samples were sonicated for 5 min in a sonication bath with ice and then centrifuged at 15,000*g* for 15 min. The supernatant was transferred to a glass tube, the pellet (including the ceramic beads) was resuspended in 500 μL of methanol, and the homogenization, sonication, and centrifugation cycle was repeated twice more to result in a three-step extraction. The total combined supernatant was concentrated to dryness under a gentle nitrogen stream and redissolved in 500 μL of methanol to a final concentration of the IS of 1 μM. Samples were stored at −20 °C until further analysis.

### 2.5 Chemical Analysis

Fluoxetine, fluoxetine-D5, and norfluoxetine concentrations were determined using a liquid-chromatography-triple quadrupole mass spectrometry system (LC-MS/MS) with ESI positive ion mode, the LCMS-8040 model (Shimadzu Corporation, Japan). Separation was performed on a UHPLC system (Shimadzu) with a Kinetex 1.7 μm C18 100 A LC column (150 mm × 2.1 mm, Phenomenex). Extraction recoveries were determined with fluoxetine-D5 (final concentration of 1 μM) and ranged from 86 to 111%, with an average of 99% (histogram in SI A2, Figure S3B). The MS/MS transitions (* used for quantification) were 310.15 > 44.1* and 310.15 > 148.2 for fluoxetine, 296.15 > 30.1 and 296.15 > 134.1* for norfluoxetine, and 314.85 > 44.1* for fluoxetine-D5. Details on the mobile phases, analysis setting, and measurement stability can be found in SI A2. To ensure stability in LC-MS/MS measurements, a calibration curve was added at the beginning and the end of each batch of samples, while after every 10 samples, a known concentration of fluoxetine-D5 was measured as an external standard (Figure S3A).

### 2.6 Gene Expression

The effects of 0, 0.5, and 20 mg/L fluoxetine on CYP gene expression over time were analyzed with quantitative real-time PCR (qRT-PCR). 1500 worms were collected in eppendorf tubes with 300 μL of RLT lysis buffer (Qiagen, Hilden, Germany) and homogenized using a Minilys homogenizer (Bertin Technologies) at middle speed four times for 20 s and kept on ice in between. Total RNA was isolated with the QIAshredder and RNeasy mini kits (Qiagen) according to the manufacturer’s protocol. The QuantiTect reverse transcription kit (Qiagen) was used for cDNA generation. RT-qPCR was performed on a Biorad CFX Opus 384 System using an iQ SYBR Green Supermix (Biorad) for amplification. The gene of interest was *cyp35-a2*, and *cdc-42* was used as a housekeeping gene. Primers were generated by Biolegio (Nijmegen, The Netherlands); further details on the RT-qPCR and primers can be found in SI A7. Three biological replicates were used for each treatment.

### 2.7 Toxicokinetic Model

**Figure 1:**
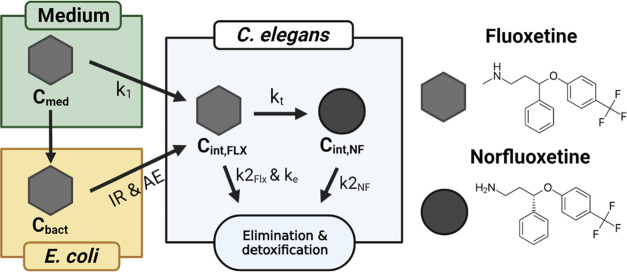
Toxicokinetic model of fluoxetine uptake by *C. elegans* from medium and *E. coli* and fluoxetine elimination and metabolism to norfluoxetine by *C. elegans*, where *k*_1_ = uptake rate constant (in L/(kg_nem_ h)), *k*_2_ = elimination rate constant (sum of *k*_2_fast__ and *k*_2_slow__) (in 1/h), *k_t_* = transformation rate constant (in 1/h), *k*_e_ = extra metabolism rate after 10 h (1/h), IR = ingestion rate (kg_bacteria_/kg_nematode_/h), AE = assimilation efficiency (0–1).

Fluoxetine uptake and norfluoxetine formation in *C. elegans* were modeled with a one-compartment model (Figure 1), using ordinary differential equations. A one-compartment model was selected since chemical concentrations could only be measured for the whole organism. Fluoxetine uptake was divided into uptake directly from the medium and uptake via *E. coli*, with norfluoxetine exclusively being formed within *C. elegans* (results in Section 3.2). Elimination was modeled as a biphasic process, as indicated in eq 1a,b. The concentration in the medium and binding to/uptake of fluoxetine in *E. coli* were found to be constant (SI A4) and also modeled that way (Figure 1). All parameters and units are summarized in SI A3.

The toxicokinetics of fluoxetine were modeled with eqs 1a and 1b
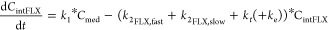
1a
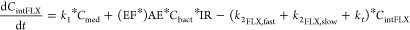
1bWith uptake rate constant *k*_1_ (L/(kg_nem_ h)), the concentration in the medium *C*_med_ (mg/L), the elimination rate *k*_2_ (1/h) (split in a fast *k*_2_FLX,fast__ and slow rate *k*_2_FLX,slow__), the transformation rate to norfluoxetine *k_t_* (1/h), the extra metabolism parameter *k*_e_ (1/h) (only used after 10 h of exposure, see the next paragraph), the internal fluoxetine concentration *C*_intFLX_ (mg FLX/kg_nem_), the assimilation efficiency AE (0–1), the concentration of *E.* coli-associated fluoxetine *C*_bact_ (mg FLX/kg_bacteria_), the ingestion rate of *E. coli* by *C. elegans* (kg_bacteria_/kg_nematode_/h), and the effect on feeding EF (0–1).

In eqs 1a and 1b, elimination was modeled as a biphasic process (with a fast *k*_2_FLX,fast__ and slow *k*_2_FLX,slow__ rate) based on the results from the elimination experiment I (Figure 3). However, during exposure, both the fast and slow eliminations will play a role simultaneously and are therefore summed. eq 1b accounts for uptake through the ingestion of *E.* coli-associated fluoxetine, as was assumed for experiments III and IV. In some cases, parameters were constrained based on outcomes of experiments I and II (see Table 3). Experimental data after 10 h of continuous exposure (for experiment IV, Figure 4C,D) revealed that the internal concentration of fluoxetine appeared to decrease over time to a new steady state. This decrease after 10 h was modeled by two different mechanisms: (1) an increase in the metabolism over time, included with an extra metabolism rate constant *k*_e_ (1/h) after 10 h in eq 1a, and (2) a decrease in feeding after 10 h with an effect of feeding EF in eq 1b. The short-term experiments (experiment II, Table 1), however, were only modeled with eq 1a, and since there was limited formation of norfluoxetine (Figure 4), the *k_t_* was not included.

The change in the internal concentration of norfluoxetine over time was modeled with eq 2
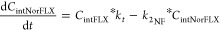
2With the internal fluoxetine concentration *C*_intFLX_ (mg FLX/kg_nem_), a constant transformation rate of fluoxetine to norfluoxetine *k_t_* (1/h), the elimination of norfluoxetine with rate constant *k*_2_NF__ (1/h) (sum of *k*_2_NF,fast__ and *k*_2_NF,slow__), and the internal norfluoxetine concentration *C*_intNorFLX_ (mg NorFLX/kg_nem_).

The kinetic bioconcentration factor^33^ was calculated with eq 3, and the *k*_e_ and *k_t_* were only included when applicable to the respective experiment
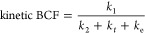
3

### 2.8 Model Fitting and Data Analysis

Model fitting was performed by adding the ordinary differential equations to the BYOM modeling package version 6.3 (http://www.debtox.info/byom.html) in Matlab R2022b.^34^ The model solves ordinary differential equations, fits parameter values, plots the observed vs predicted response, and creates parameter likelihood plots with 95% confidence intervals. Optimization was performed with the Melder–Mead Simplex Search method, minimizing the minus log-likelihood. Reasonable initial values were estimated in Microsoft Excel; five sets of initial values between 0.1 and 10 were used to check the effect of initial values on parameter fitting, and the values that resulted in the lowest AIC were selected. Model script deviations from the BYOM model can be found in SI A9.

For all data points, the mean and standard deviation were plotted using Graphpad Prism 9.4. The ordinary differential equations and parameters fitted in Matlab were also entered in Graphpad Prism to simulate the internal concentrations over time. For the behavioral effects, mono-, bi-, and triphasic nonlinear regression models were applied in Graphpad Prism and their AICc values were compared to determine the best fit.^14,35^ Significant differences in toxicity between fluoxetine and norfluoxetine were tested with a two-way ANOVA, and Bonferroni post hoc test was applied with a *p*-value <0.05. The kinetic BCF, *k*_1_, and *k*_2_FLX,sum__ were also plotted against the medium concentrations in Graphpad Prism.

## 3 Results and Discussion

### 3.1 Relative Toxicity of Fluoxetine and Norfluoxetine

**Figure 2:**
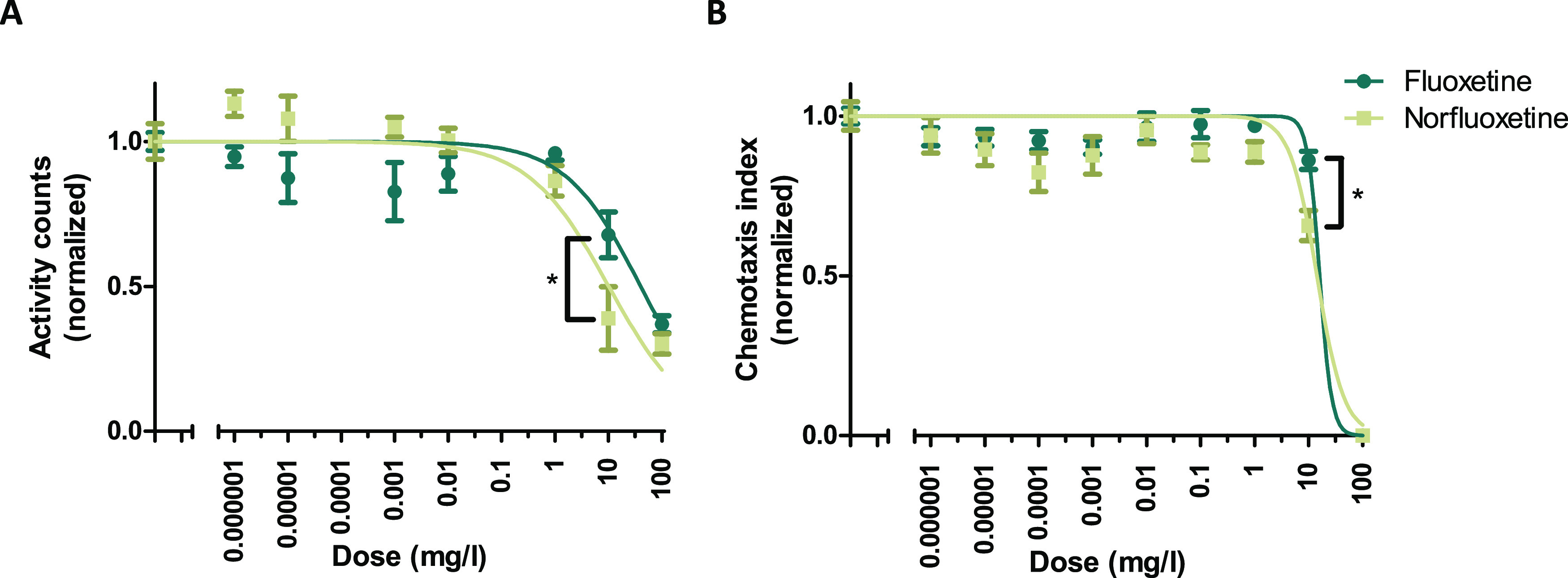
Effect of fluoxetine and norfluoxetine on *C. elegans* (a) activity and (b) chemotaxis behavior. Mean ± SEM is represented, and brackets with * indicate *p* < 0.05 between fluoxetine and norfluoxetine at that concentration level.

Effects of fluoxetine and norfluoxetine on *C. elegans* activity and chemotactic behavior were observed (Figure 2). The two compounds induced similar dose–response patterns, but a two-way ANOVA indicated significant effects of both concentration (*p* < 0.001) and compound (*p* = 0.0018). Only at 10 mg/L, norfluoxetine exposure resulted in a significantly lower activity and chemotaxis index compared to fluoxetine (*p* < 0.01 with Bonferroni post hoc analysis). The respective EC50s (+95% confidence intervals) for the effect on activity were 40.0 (11.4–68.5) mg/L for fluoxetine and 10.1 (3.18–17.0) mg/L for norfluoxetine and for chemotaxis 16.0 (−56.9 to 88.9) mg/L for fluoxetine and 14.22 (8.64–19.80) mg/L for norfluoxetine. So, there is an indication that norfluoxetine is slightly more potent. This comparison is based on nominal concentrations, but the internal concentrations of norfluoxetine were found to be around 70% compared to fluoxetine for the same exposure concentration (SI A5, Figure S9), also indicating higher potency. It is important to acknowledge, however, that toxicity is based on concentrations at the actual target site, which we could not measure in this experiment. However, this potency of norfluoxetine does imply that, depending on its internal concentration and thus on its kinetics for formation and elimination, norfluoxetine might contribute to the toxicity of fluoxetine. The dose–response curves also suggest a nonmonotonic dose–response relationship, especially for chemotaxis, as was observed before for fluoxetine exposure of *C. elegans*(14) and other species.^6,36−39^

### 3.2 Medium and *E. coli* Concentrations

**Figure 3:**
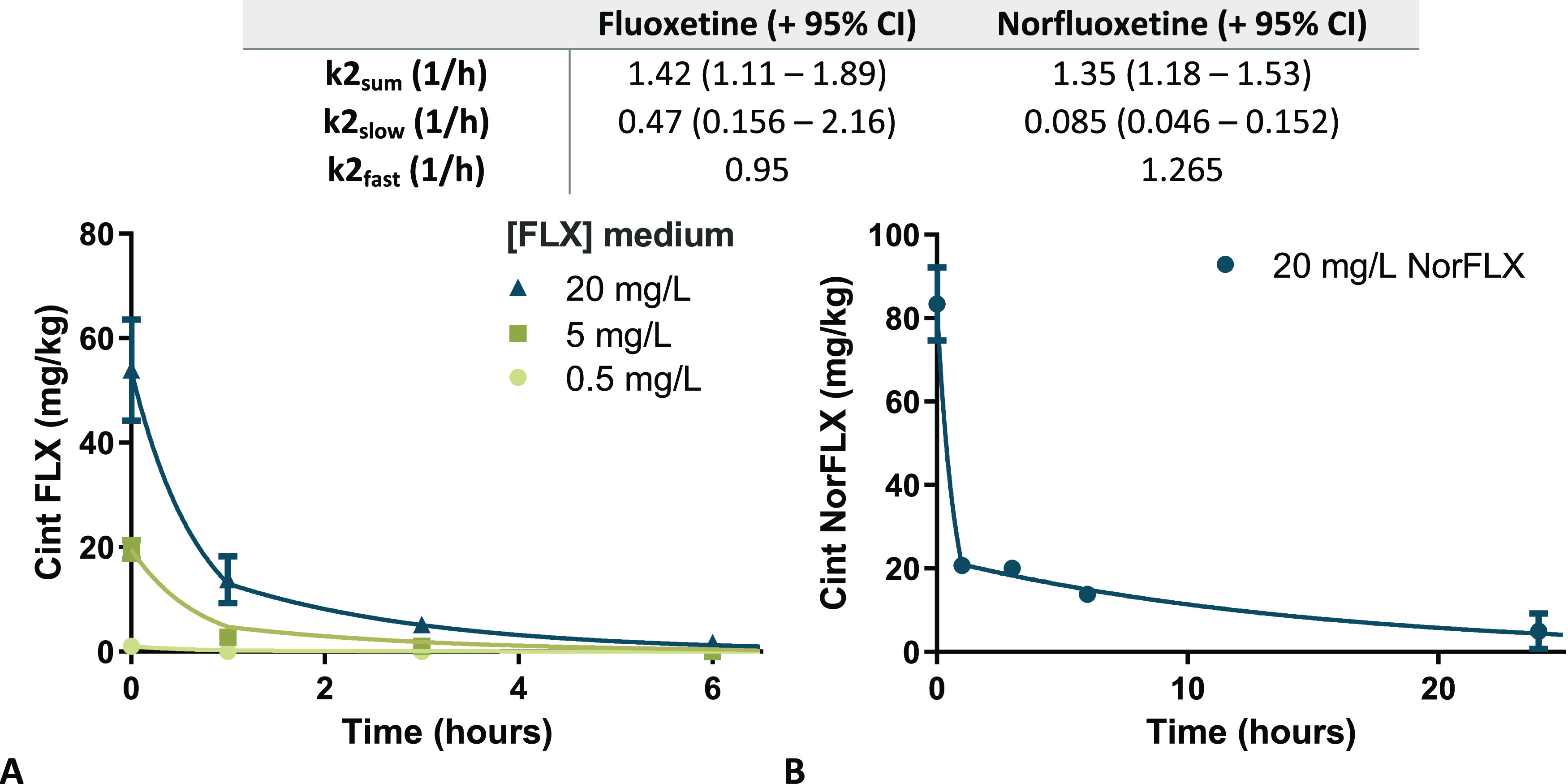
Elimination of fluoxetine (a) and norfluoxetine (b) by *C. elegans*. Nematodes were exposed for 1 h to 0.5, 5, or 20 mg/L fluoxetine and to 20 mg/L norfluoxetine and then washed and added to a clean medium to measure internal concentrations after 1, 3, 6, and 24 h. Best-fit parameter values of elimination rates are given in Table 2.

**Figure 4:**
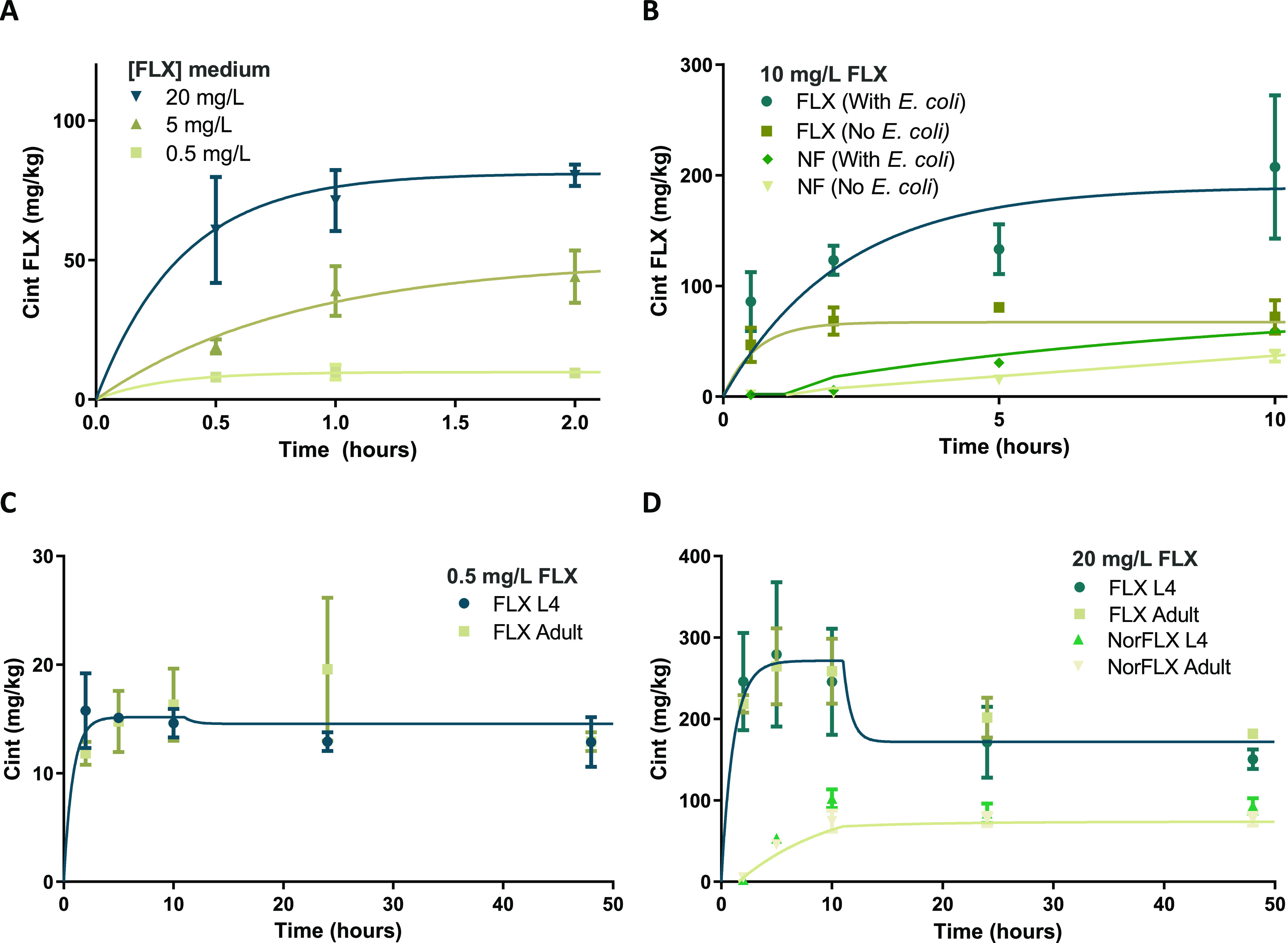
Internal concentrations of fluoxetine by *C. elegans* for (A) exposure to 0.5, 5, and 20 mg/L for 2 h (experiment II) and (B) exposure to 10 mg/L for 10 h (experiment III) and exposure for 48 h to 0.5 mg/L (C) and 20 mg/L (D). Best-fit parameter values for uptake and elimination are shown in Table 3. Details of the experiments can be found in Table 1. Bars represent the mean ± the SD as calculated with three independent replicates.

The concentration of fluoxetine in S medium without *C. elegans* was found to be constant over time (SI A4, Figure S4). The concentration in medium was lower when bacteria were copresent in the medium at a high optical density at 600 nm of 1.0, suggesting that fluoxetine was either bound to or taken up by the *E. coli* (Figures S4 and S5). *E. coli* can thus affect the bioavailability of fluoxetine, but lower concentrations of bacteria were used in the experiments with *C. elegans*. Furthermore, medium concentrations were also measured during the experiments to account for this. Norfluoxetine concentrations in both medium and bacteria exposed to fluoxetine were negligible; therefore, if norfluoxetine was found in *C. elegans*, this was caused by metabolism in the nematode. The concentration of fluoxetine in *E. coli* OP50 (SI A4, Figure S5) was significantly higher for not-washed bacteria compared to that for washed bacteria, which indicates that fluoxetine is adsorbed to the outside of *E. coli*. The toxicokinetic model assumed the concentration for the not-washed bacteria (*C*_bact_ in eq 1b) because the fluoxetine adsorbed to the outside of the bacteria will also be taken up by *C. elegans*. The actual concentrations in the medium were also quantified for all experiments (SI A4, Figure S7 and Table S4). Measured concentrations were slightly higher than nominal concentrations, and the amount adsorbed to and taken up by bacteria was only a small fraction of the total mass. Figures 3 and 4 report the nominal concentrations, but measured concentrations (Table S4) were used for the toxicokinetic modeling, and the reported parameters (*k*_1_, *k*_2_FLX,sum__, *k*_e_, *k_t_*, AE, EF) were thus obtained by fitting the data using measured medium concentrations.

### 3.3 Concentration-Dependent Uptake and Elimination

*C. elegans* has an excretory system that is somewhat similar to the human renal system, and phase I and II enzymes are present in many human orthologs.^21^ The elimination of fluoxetine and norfluoxetine, measured after loading with *C. elegans* via preincubation with the respective model compounds, is illustrated in Figure 3. The concentrations of fluoxetine and norfluoxetine in *C. elegans* decreased rapidly in the first hour after transfer to a clean medium (Figure 3), but the rate of elimination decreased over time. These differences in elimination rate were best modeled with a fast and a slow *k*_2_ (Table 2). The TK model assumed a switch to a slow elimination rate at 1 h, but due to a lack of data between 0 and 1 h, it was not possible to estimate the exact time point, which may actually be somewhat earlier. *C. elegans* is known to depurate its gut within minutes,^40^ so the initial drop over the first hour of incubation is probably not related to this process. Likely, part of the fluoxetine and norfluoxetine will be more readily available for elimination than another part that may be bound. A similar pattern of elimination has been observed in *C. elegans* for chlorpyrifos^22^ and phenanthrene elimination.^23^ Spann et al. addressed this by adding to the kinetic model a peripheral compartment, apart from a central compartment, where a compound can be bound and where elimination is slower than from the central compartment.^23^ However, in *C. elegans*, it is difficult to estimate the amount of a chemical in each compartment, so therefore, we chose to model a single compartment with a slower and a faster elimination rate constant over time to describe this biphasic elimination. While almost all fluoxetine was eliminated after 6 h, norfluoxetine was still present in small amounts after 24 h. This longer elimination half-life of norfluoxetine as compared to that of fluoxetine has also been observed in humans.^41^ Elimination rates were not dependent on the exposure concentration of fluoxetine, and while we tested only one concentration of norfluoxetine, we assume the same.

**Figure 5:**
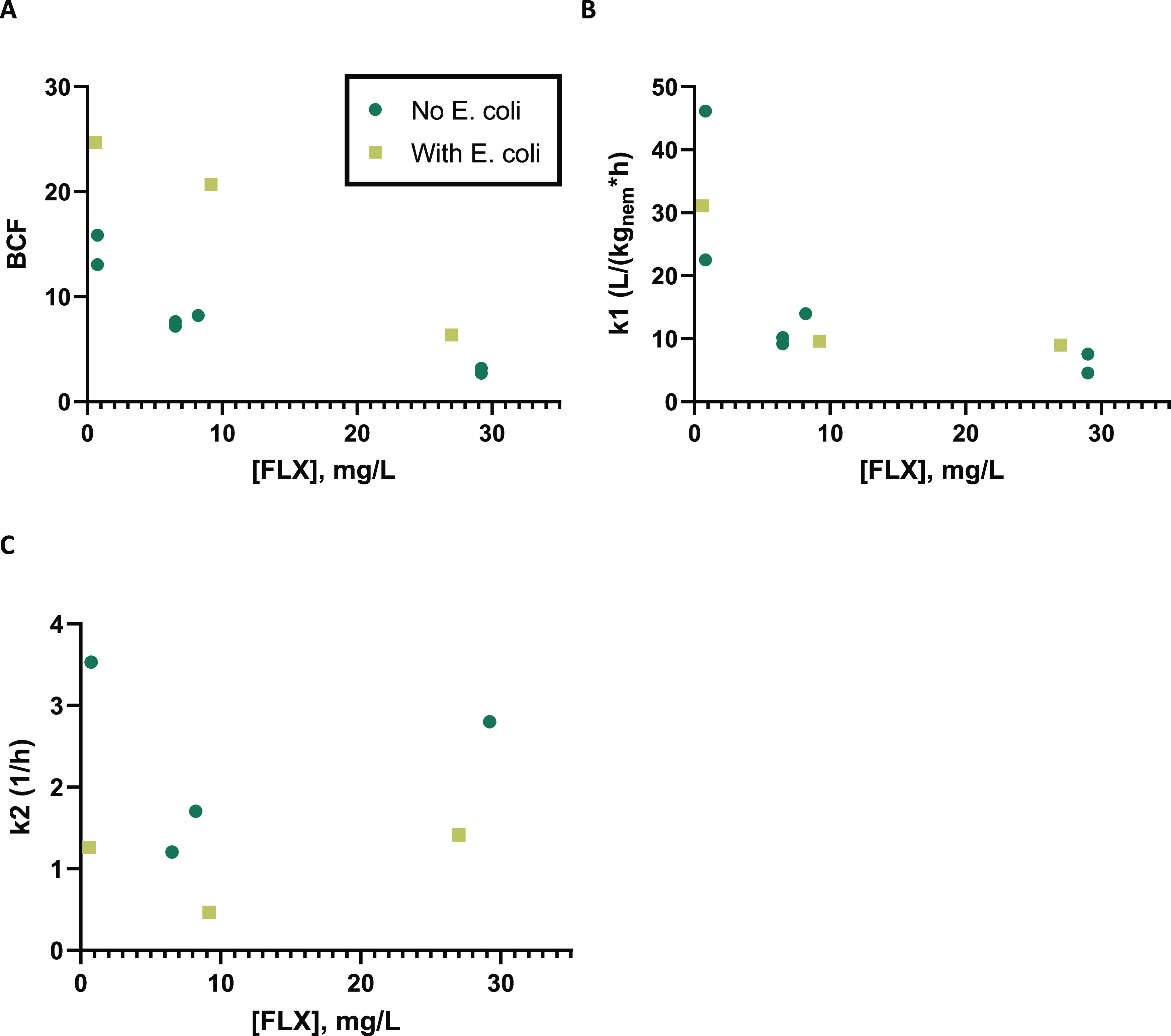
Fluoxetine concentration (*C*_med_)-dependent values for toxicokinetic parameters: (A) kinetic bioconcentration factor (BCF), (B) uptake rate constant (*k*_1_), and (C) elimination rate constant (*k*_2_FLX__ + *k_t_* + *k*_e_) combined from experiments II, III, and IV (Table 1).

*C. elegans* has two potential uptake routes for chemicals: through the cuticle and through the pharynx, which can both act as a strong barrier.^21,26^ These barriers may result in the limited uptake of chemicals, which is sometimes regarded as a drawback of using *C. elegans* as a model species in high-throughput toxicity assays.^21^ However, the current study shows that fluoxetine was taken up by *C. elegans* and also metabolized into its active metabolite norfluoxetine (Figure 4). The internal concentrations of fluoxetine increased for higher-medium concentrations (Figure 4A), and the bioconcentration and uptake parameter *k*_1_ also appeared to be concentration-dependent (Figure 5). A similar concentration-dependent trend in BCF was observed in studies with fluoxetine using marine mussels,^42^ freshwater mussels,^43^*Daphnia magna,*^44^ and zebrafish.^45^ The increase in the BCF for lower exposure concentrations also indicates that the BCF is likely even higher for more environmentally relevant lower exposure concentrations, but testing this would require a high amount of biological material. The previously mentioned studies with other species have found relatively higher BCF values, varying from 100 to 10,000.^42−44,46^ This relatively low BCF for *C. elegans* could be related to the often discussed strong barriers for uptake in the cuticle and the pharynx. Differences in body composition can also play a role, but comparing *C. elegans* body composition (dry biomass with around 60% protein and 20% lipids)^47^ to that of *D. magna* (82.5% protein and 6.7% lipids)^48^ does not give a clear indication as to why *C. elegans* might have lower BCF values. Furthermore, a recent study showed that organism lipid contents were not positively correlated with BCF/BAF values for SSRIs for four different species, indicating that other factors might be more important.^49^ As indicated before, BCF values in this study could also be affected by saturation since relatively high concentrations were used, so BCF values should be interpreted with care.

*C. elegans* in this study has been shown to have a relatively fast elimination rate of fluoxetine. The *k*_2_ values for fluoxetine for the crustacean *Gammarus pulex*, Nile tilapia, and Japanese Medaka were found to be much lower, in the range of 0.0008–0.08/h.^50−52^ In general, a fast elimination rate could be explained by higher metabolic rates, a fast excretion pathway, or related to lower binding capacities because tissue incorporation is known to affect elimination and therefore bioaccumulation.^49^ However, not much is known about these aspects of *C. elegans* elimination and this would be an important area of future research to be able to better examine the suitability of *C. elegans* as a model organism for ecotoxicology.

*C. elegans* fluoxetine concentrations were found to rapidly increase and reach peak concentrations after 2–5 h (Figure 4). Already from 0.5 to 1 h, the uptake is nonlinear, so it was not possible to fit the *k*_1_ independent of the *k*_2_FLX,sum__ and both were fitted simultaneously according to eq 1a. Since the *k*_2_FLX,sum__ was already determined independently (Figure 3 and Table 2), this value was also included in the model, fitting just *k*_1_ (Table 3). The model performed slightly better when the *k*_2_FLX,sum__ was not specified *a priori*, as can be seen from the slightly different *R*^2^-values in Table 3 for some of the models. Previous studies into *C. elegans* toxicokinetics have found similar rapid uptake for chlorpyrifos^22^ and ethanol,^19^ while for some other compounds, such as phenanthrene, methyl mercury, and cadmium, the uptake was slower, i.e., still increasing for phenanthrene after 24 h and methyl mercury after 15 h.^17,18,23,24^ Phenanthrene has a much higher lipophilicity, which is often linked to a slower elimination rate, explaining the longer time to reach steady state.^23^

**Table 2:** Best-Fit Parameter Values for Elimination Rate Constants (1/h) for Fluoxetine and Norfluoxetine Elimination by *C. elegans* as Derived from the Kinetic Data in Figure 3^a^

	fluoxetine (+95% CI)	norfluoxetine (+95% CI)
*k*_2_sum__ (1/h)	1.42 (1.11–1.89)	1.35 (1.18–1.53)
*k*_2_slow__ (1/h)	0.47 (0.156–2.16)	0.085 (0.046–0.152)
*k*_2_fast__ (1/h)	0.95	1.265

a *k*2_fast_ was calculated from the values of *k*_2_sum__ and *k*_2_slow__.

**Table 3:** Parameters for Modeling of Experiments II, III, and IV Using Eqs 1a,1b–3^a^

exp	*E. coli*?	conc. (mg/L)	time	constr.	*k*_1_	95% CI	*k*_2_f__	95% CI	*k_t_*	95% CI	*k*_2_NF__	95% CI	others	95% CI	BCF FLX	*R*^2^ FLX	*R*^2^ NF
II	no	0.5	2 h		46.1	29.3–100	3.53	2.06–8.69							13.06	0.91	
II	no	0.5	2 h	*k*_2FLX_	22.5	19.3–25.8	*1.42*	1.07–10.3							15.85	0.81	
II	no	5	2 h		9.19	6.17–13.9	1.2	0.56–2.17							7.66	0.89	
II	no	5	2 h	*k*_2FLX_	10.2	8.95–11.4	*1.42*	0.54–2.24							7.18	0.89	
II	no	20	2 h		7.58	5.01–16.2	2.8	1.67–6.54							2.71	0.92	
II	no	20	2 h	*k*_2FLX_	4.54	4.00–5.08	*1.42*	1.08–47.4							3.20	0.87	
III	no	10	10 h		14	8.40–22.5	1.66	0.93–2.81	0.05	0.045–0.060	0.0001	<0.001–0.038			8.19	0.89	0.96
III	yes	10	10 h		9.61	4.83–21.0	0.4	0.052–1.13	0.065	0.042–0.12	0.12	0.017–0.36			20.67	0.71	0.99
III	yes	10	10 h	*k*_1_, *k*_2FLX_, *k*_2_NF__, *k_t_*	14	<0.001–24.2	1.66	0.78–2.28	0.05	0.036–0.063	0.0001	<0.03–0.081	IR = 0.098	0.088–0.11	8.19	0.62	0.99
IV	yes	0.5	48 h		31.1	17.0–100	1.21	0.62–4.29					ke = 0.051	<0.001–0.60	24.66	0.78	
IV	yes	20	48 h		9.01	6.13–13.6	0.84	0.52–1.36	0.056	0.048–0.070	0.11	0.091–0.16	ke = 0.52	0.28–0.82	6.36	0.84	0.93

a Experimental details for experiments II–IV can be found in Table 1. Column 5 (fix) indicates which one of the parameters was constrained for model fitting.

### 3.4 Effect of *E. coli*: Quantifying Different Routes of Uptake

Internal concentrations of fluoxetine in *C. elegans* were significantly higher in the presence of *E. coli* OP50 (Figures 4B and 5). Such higher internal concentrations in the presence of *E. coli* have been found before for phenanthrene^23^ and iron nanoparticles.^24,25^ Interestingly, the presence of bacteria almost doubled the internal concentration of fluoxetine in experiment III (for 10 mg/L), similar to what was found for phenanthrene.^23^ The effect of *E. coli* (Figure 4B) was modeled with both eqs 1a and 1b. For eq 1b that accounts for the ingestion of *E. coli*, the *k*_1_, *k_t_*, *k*_2_FLX__, and *k*_2_NF__ were constrained to those of the same exposure concentration without *E. coli* in order to just model the effect of the ingestion of bacteria-associated fluoxetine. However, in order for eq 1b to explain the difference in internal concentrations, the ingestion rate has to be 0.098 (95% CI 0.088–0.109) kg_bact_/kg_nem_/h, which is almost seven times higher than the ingestion rate of 0.0142 as suggested by others.^23,25^ Therefore, the ingestion of *E. coli*-associated fluoxetine in itself does not explain the differences in internal concentrations. An increase in pharyngeal pumping might also be associated with an augmented intake of liquid, although alterations in the body composition are likely to be a contributing factor.

Figure 5 shows that *k*_1_ was not so dependent on the presence of bacteria, while *k*_2_FLX,sum__ actually decreased to around half. A smaller *k*_2_FLX,sum__ resulted from the fact that it took longer for the internal fluoxetine concentrations to reach a steady state when *E. coli* was present, meaning that the increased internal concentrations are more likely linked to a decrease in the elimination of fluoxetine than to an increase in the uptake. Spann et al. relate the decreased elimination rate of phenanthrene to higher lipid levels in *E. coli*-rich exposure scenarios. Since *C. elegans* was found to be able to decrease its total amount of lipids to 50% within 6 h,^21,23^ this could also play a role in this study. However, former studies showed that the organism lipid content did not predict SSRI accumulation well,^49^ so the extent to which this process plays a role should be further investigated for *C. elegans*. In general, fluoxetine taken up through ingestion via the intestine may also be better integrated than that taken up through diffusion through the skin, potentially leading to different elimination rate constants for chemicals accumulated via the different exposure routes.

### 3.5 Formation and Relative Contribution to Biological Effects of Norfluoxetine

Norfluoxetine is known to be the major active metabolite of fluoxetine, but there are also other metabolites and both fluoxetine and norfluoxetine undergo glucuronidation.^53^ The current model does not account for the other metabolites, but because *k*_2_ describes general clearance, it includes those as well. Norfluoxetine was detected in *C. elegans*, with an increase in internal concentrations over time (Figure 4B–D). Since norfluoxetine was not detected in *S* medium or *E. coli* without nematodes present, this means that biotransformation occurred within *C. elegans*. Norfluoxetine formation was modeled with a transformation rate *k_t_* of around 0.06 (1/h) and with an elimination rate (*k*_2_NF__) of around 0.12/h. For 20 mg/L, norfluoxetine concentrations reached a steady state after 10 h at a concentration of approximately 80 mg/kg_nem_, around half of the fluoxetine concentrations at a steady state (Figure 4D). With its comparable or even higher potency, longer elimination half-life, and substantial internal concentrations in the same order of magnitude as those of fluoxetine, norfluoxetine can clearly contribute to the toxicity of fluoxetine. The importance of accounting for pharmaceutical transformation products in toxicological research was also pointed out by a recent review, which identified 98 active pharmaceutical transformation products, including norfluoxetine.^27^ However, while the toxicity of fluoxetine has been widely studied, to the best of our knowledge, only a few studies explicitly consider the ecotoxicological effects of norfluoxetine.^54−60^ This may be even more important, knowing that norfluoxetine has been detected in influent, effluent, and freshwater and the environmental exposure distribution indicated a higher abundance of norfluoxetine than of fluoxetine.^61^

Norfluoxetine was also detected in the exposure medium for experiments III and IV and increased over time, indicating the elimination of norfluoxetine. In terms of mass, this is only limited compared to internal concentrations of fluoxetine and norfluoxetine, and therefore, this was not considered in the model. However, this does allow for a calculation of the excretion rate of norfluoxetine, as shown in SI A6. The calculated excretion rate was found to be around 0.1 (/h) for 0.5 and 10 mg/L of fluoxetine, which was between the confidence intervals for the fitted *k*_2_NF__ (Table 3). However, for 20 mg/L of fluoxetine exposure, the calculated excretion rate was around 0.5/h and even increased to 0.83, outside of the 95% CI for the estimated *k*_2_NF__ of 0.091–0.158. This could be explained by the saturation of binding sites for norfluoxetine at these higher concentrations, but this still requires further investigation.

### 3.6 Long-Term Dynamics in Internal Concentration

Long-term (48 h) exposure of *C. elegans* to fluoxetine was performed (Figure 4C,D) since this is a timespan more similar to previously performed toxicity assays with *C. elegans*.^14^ Interestingly, between 10 and 24 h, there was a decrease in the internal concentration of fluoxetine to a new steady state (Figure 4D), even though concentrations in the medium did not decrease (SI A4). Different theories could explain this pattern. Because *C. elegans* was exposed at the L4 stage, ecdysis and cuticle structure differences when molting to the adult stage could decrease the chemical content in the body. However, exposure experiments with L4 and adult *C. elegans* did not show significant differences in steady-state concentrations between the two (Figure 4C,D). Another explanation would be that the formation of norfluoxetine over time might compete for the binding sites of fluoxetine, as was observed for fluoxetine and cortisol binding to human serum albumin,^62^ resulting in a decrease in internal fluoxetine concentrations by making fluoxetine more bioaccessible for elimination. To test this, *C. elegans* was coexposed to fluoxetine (5 and 20 mg/L) and norfluoxetine (20 mg/L) (SI A5, Figure S8), but no significant differences were found in internal fluoxetine concentrations, even though the uptake of norfluoxetine was confirmed (Figure S9). A toxic effect on feeding could also result in lower chemical uptake since previous studies found an EC50 for the effect of fluoxetine on *C. elegans* feeding behavior of 7 mg/L. This was modeled with eq 1b, but this could only explain a very small fraction of the decrease. Alternatively, there could be a concentration-dependent increase in metabolism over time resulting from enzyme induction. This was modeled with an extra metabolism rate constant ke that was included after 10 h (eq 1a and Figure 4C,D). The ke value was an order of magnitude higher for 20 mg/L compared to that for 0.5 mg/L, which would imply a concentration dependency of this phenomenon, which is not unlogic given that the conversion of fluoxetine to norfluoxetine is mediated by cytochrome P450.^63,64^ Gene expression results (SI A7, Figure S11) show an increase in CYP gene expression over time for 20 mg/L of fluoxetine exposure, while this increase was not observed for 0.5 mg/L. The increase was also only observed for 24 h and not yet for 10 h after exposure, which is consistent with the pattern observed in Figure 4D. Therefore, the increase in metabolism seems to be the most likely explanation, but an effect on feeding and pharyngeal pumping could be simultaneously involved.

### 3.7 Parameter Fitting and Model Uncertainty

Parameter fitting was performed with the Melder–Mead Simplex Search method, minimizing the minus log-likelihood. The uptake rate (*k*_1_), elimination rate (*k*_2_FLX,sum__), transformation rate (*k_t_*), and extra metabolism rate (*k*_e_) or assimilation efficiency (AE) and feeding effect (EF) constants were fitted simultaneously. The *k*_1_ could not be fitted individually because internal concentrations reached a steady state rapidly. Because of this simultaneous fitting, there is a wide range in some of the confidence intervals. This could be improved by constraining the *k*_2_FLX,sum__ based on the elimination experiment, but in some cases, this decreased the goodness of fit. SI A8 shows the parameter space plots for all fitted parameters and indicates that even with the wide confidence intervals, the parameters were clearly fitted at the lowest log-likelihood.

### 3.8 Implication of the Study

The ability of *C. elegans* to accumulate and metabolize compounds was found to be limited for many substances.^26^ However, this study confirmed that these kinetic processes take place for fluoxetine. Given that norfluoxetine exhibits a somewhat higher potency, a longer elimination half-life, and reaches internal concentrations in the same order of magnitude as fluoxetine, this metabolite will have a substantial contribution to the behavioral toxicity of fluoxetine in *C. elegans*. Whether this also holds for other species remains to be established and is dependent on their metabolic profiles. Prior studies already suggested to additively take into account the presence of metabolites in exposure and effect assessment of pharmaceuticals in the aquatic environment.^65^ The BCFs for fluoxetine obtained in the present study for *C. elegans* were relatively low compared to those obtained for other organisms in other studies,^42−44,46^ confirming the importance of accounting for the toxicokinetics when comparing species sensitivities and determining relevant toxic concentrations. The BCFs should be interpreted with caution, as they could increase for lower concentrations due to saturation. The BCFs and uptake and elimination rate constants were dependent on the fluoxetine concentration in the medium (Cmed), exposure duration, and presence of bacteria. The context-dependent nature of these kinetic rates underscores the importance of considering such factors in future studies and risk assessment. Outcomes obtained at one concentration or time frame cannot directly be extrapolated to other concentrations, especially not to environmentally relevant concentrations. Table 4 summarizes key factors influencing toxicokinetics, as discovered in this study.

**Table 4:** Factors Possibly Influencing the Toxicokinetics in *C. elegans* and the Effects on the TK of Fluoxetine Based on the Results of This Study

factor	effect on TK of fluoxetine	other *C. elegans* studies on this
exposure concentration	BCF: negatively correlated	
	*k*_1_: negatively correlated	
presence of *E. coli*	BCF: positively correlated	(23,24,66)
	*k*_2_: negatively correlated	
body composition—lipids and proteins (*E. coli* related)	not tested but discussed based on former studies	(21,23)
(toxic) effect on feeding (*E. coli* related)	IR (ingestion rate): negatively correlated–neurotoxic effect	
CYP P450 induction (linked to exposure time)	*k*_2_: positively correlated (adding extra ke parameter over time)	(22)
coexposure with norfluoxetine	tested, but no effect	(21,67)
life stage	tested, but no effect (L4 vs adult)	(21)

Some questions related to the toxicokinetics of fluoxetine still remain to be answered. Both fluoxetine and norfluoxetine have an *R*- and an *S*-enantiomer that vary in potency.^68−70^ The racemate is used for therapeutical purposes,^71^ but binding or metabolism might be stereoselective, and this should be further investigated to better predict toxicity in an environmental setting. Furthermore, it would be interesting to test for fluoxetine and norfluoxetine bioaccumulation at even lower external medium concentrations, similar to environmental concentrations in the range of ng/L.^6,36−39^ However, this would require an unrealistically large amount of tissue. Overall, the characterization of fluoxetine uptake and norfluoxetine formation in *C. elegans* as done in the current study can clearly contribute to explaining and predicting the dynamics of neurotoxic and behavioral effects of this antidepressant.

## Supporting Information Available

Additional experimental details for behavioral tests and chemical analysis, further model descriptions, data on concentrations of fluoxetine in medium and bacteria, data on the uptake of norfluoxetine, *cyp35-a2* gene expression data and primer information, model parameter space plots, and MATLAB scripts (PDF)
