# Supplementary material for: Toxicokinetics of the Antidepressant Fluoxetine and Its Active Metabolite Norfluoxetine in Caenorhabditis elegans and Their Comparative Potency
Source: Environ Sci Technol. 2024 Feb 12;58(7):3129–40. doi: 10.1021/acs.est.3c07744 (PMC10882974; doi:10.1021/acs.est.3c07744)
Supplement: Supplementary file 1 — es3c07744_si_001.pdf [file es3c07744_si_001.pdf]

Supplementary material to 'Toxicokinetics of the antidepressant fluoxetine and its active metabolite norfluoxetine in *Caenorhabditis elegans* and their comparative potency '

Journal: Environmental Science and Technology

Authors: Merel A. van der Most*, Wouter Bakker, Sebastiaan Wesseling and Nico W. van den Brink

Division of Toxicology, Wageningen University and Research, Wageningen 6708 WE, the Netherlands
Corresponding author (indicated by *): *merel.vandermost@wur.nl*

Summary: 17 pages, 11 Figures, 5 Tables

Content:

SI A1: Experimental details for behavioural experiments with C. elegans

SI A2: Chemical analysis details, including the LC-MS/MS method and extraction recoveries

SI A3: Model parameters, states, their units and descriptions

SI A4: Concentrations of fluoxetine and norfluoxetine in medium and bacteria

SI A5: Uptake of norfluoxetine in C. elegans compared to fluoxetine

SI A6: Medium concentrations of norfluoxetine and excretion rates over time

SI A7: Changes in cyp35a2 gene expression over time upon fluoxetine exposure

SI A8: Parameter space plots used to determine confidence intervals around model parameters

SI A9: MATLAB model scripts based on BYOM modelling package: derivatives

SI A1: Experimental details for behavioural experiments with C. elegans

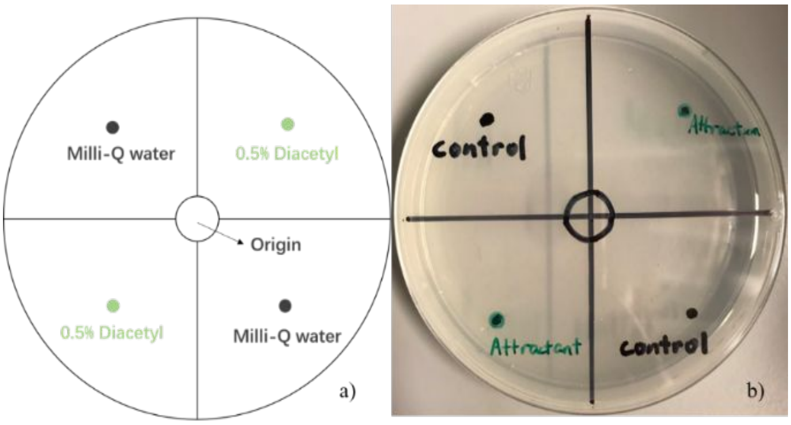

Figure S1: Set-up of the chemotaxis index experiments with C. elegans, using a Ø10cm petri dish with nematode growth medium, radius of origin 0.5cm

The chemotaxis index was calculated with the following equation:

$$Chemotaxis\ index = \frac{\#worms\ in\ quadrants\ 'attraction' - \#worms\ in\ quadrants\ 'control'}{Total\ \#worms} \quad (Eq\ 1)$$

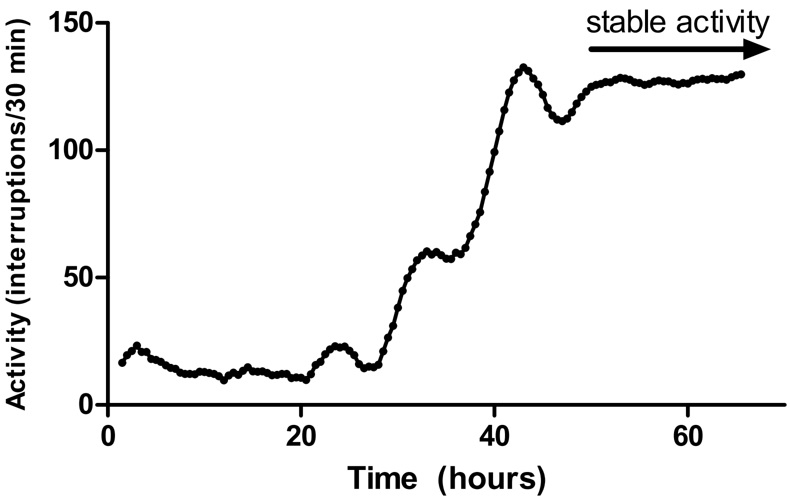

Figure S2: Development of C. elegans over time expressed as activity, measured as interruption counts per 30 minutes with the WMicrotracker. Chemotaxis and feeding were tested for 72h starting exposures at L1 larvae and activity was measured for 21h after a basal measurement of stable activity at around 50 hours. (based on Van der Most et al. 2023)

SI A2 : Chemical analysis details, including the LC-MS/MS method and extraction recoveries

Table S1: LC-MS/MS method – mobile phase details

	(A) MiliQ with 0.01% formic acid	(B) Acetonitrile
**0 - 1 min**	100	0
**1 - 3 min**	to 0	to 100
**3 - 9 min**	0	100
**9 - 9:30 min**	to 100	to 0
**9:30 - 15 min**	100	0

Table S2: LC-MS/MS method – instrument and method details

**Model**	LCMS-8040 model (Shimadzu Corporation, Japan)
**Column**	Kinetex 1.7 µm C18 100 A LC column (150 x 2.1 mm, Phenomenex)
**Mode**	ESI positive ion mode
**Sample injected**	1 µl
**Flow rate**	0.2 ml/min.
**Column temperature**	40°C
**Sample tray temperature**	4°C
**Nebulizing gas flow**	3 L/min
**Drying gas flow**	15 L/min
**DL temperature**	250°C
**Heat block temperature**	400 ^{o}C
**Detection mode**	Multiple reaction monitoring (MRM)
**Mass transitions**	310.15>44.1* and 310.15>148.2 for fluoxetine, 296.15>30.1 and 296.15>134.1* for norfluoxetine and 314.85>44.1* for fluoxetine-D5

A)

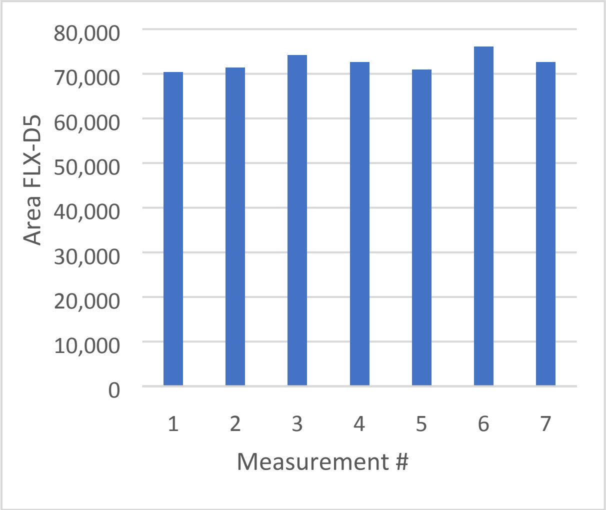

B)

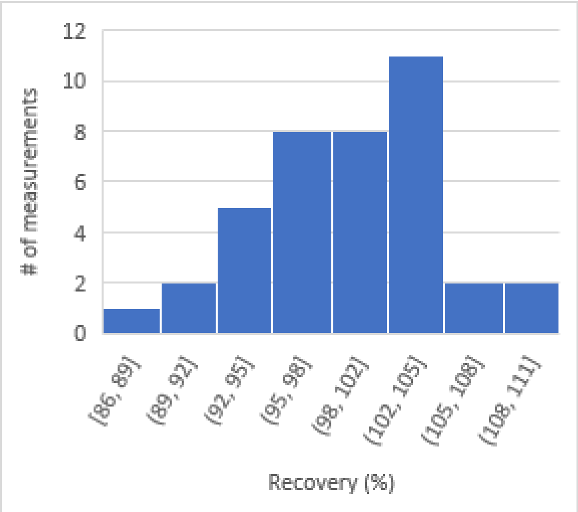

Figure S3: A) Stability over time with LC-MS internal standard every 10 measurements, example from experiment III and B) Fluoxetine-D5 recoveries in worm samples, example from experiment III

SI A3: Model parameters, states, their units and descriptions

Table S3: Parameters used for the one-compartment model

**Parameter**	**Unit**	**Description**
$k2_{FLX,fast}$	(1/h)	The fast elimination rate constant of fluoxetine. Elimination is the combination of excretion and metabolism (excluding the biotransformation to norfluoxetine)
$k2_{FLX,slow}$	(1/h)	The slow elimination rate constant of fluoxetine
$k2_{FLX}$	(1/h)	The sum of $k2_{FLX,fast}$ and $k2_{FLX,slow}$
$k2_{NF,fast}$	(1/h)	The fast elimination rate constant of norfluoxetine
$k2_{NF,slow}$	(1/h)	The slow elimination rate constant of norfluoxetine
$k2_{NF}$	(1/h)	The sum of $k2_{NF,fast}$ and $k2_{NF,slow}$
k1	$(L/(kg_{nem}*h))$	The uptake rate constant of fluoxetine by *C. elegans* from the medium
kt	(1/h) = $(mg_{nf}/kg_{nem})/(mg_{flx}/kg_{nem})/h$	The biotransformation rate constant of norfluoxetine from internal fluoxetine concentrations
AE	(0 - 1)	The efficiency with which fluoxetine was assimilated from the *E. coli* food source
IR	kg bact/kg nem/hour	The ingestion rate (mass of bacteria consumed per kilogram *C. elegans* per hour), as determined to be 0.0142 based on wet mass by Spann et al. (2015)
ke	(1/h)	An extra elimination rate constant over time
EF	(0 - 1)	The behavioural effect of fluoxetine on the ingestion rate of bacteria by *C. elegans*
**States**		
$C_{int,FLX}$	mg flx/kg nem	*C. elegans* internal fluoxetine concentration
$C_{int,NF}$	mg nf/kg nem	*C. elegans* internal norfluoxetine concentration
C_{med}	mg flx/L med	Fluoxetine concentrations in the liquid medium
C_{bact}	mg flx/kg bact	The total concentrations of fluoxetine in *E. coli,* both taken up and bound

SI A4: Concentrations of fluoxetine and norfluoxetine in medium and bacteria

Concentrations in medium were found to be stable over a 24 hour time period in S medium (Figure S5). Concentrations when bacteria at an optical density of 1.0 were added, were slightly lower. The amount of fluoxetine bound to bacteria (not washed, Figure S6), actually contributed to the difference in medium concentrations, as seen in the mass balances in Figure S7.

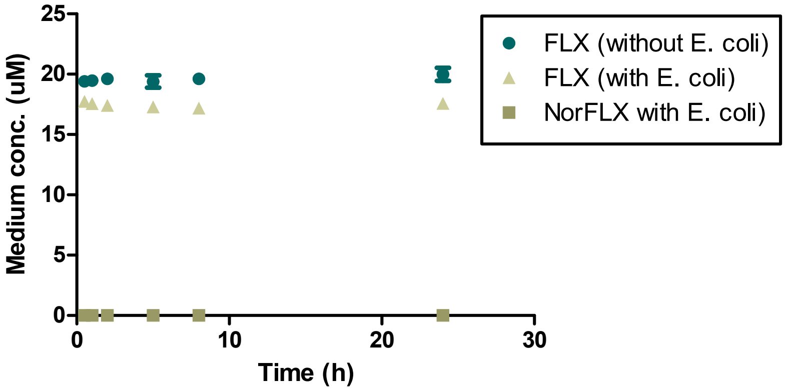

Figure S4: Concentration of fluoxetine and norfluoxetine in S medium over time

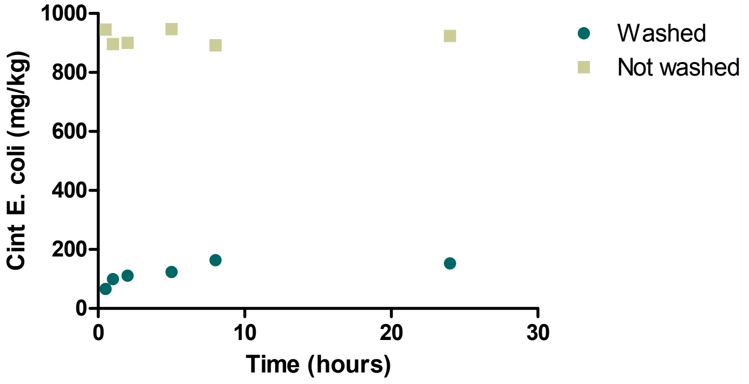

Figure S5: Concentrations of fluoxetine in *E. coli* OP50 over time, exposure to 10 mg/L fluoxetine

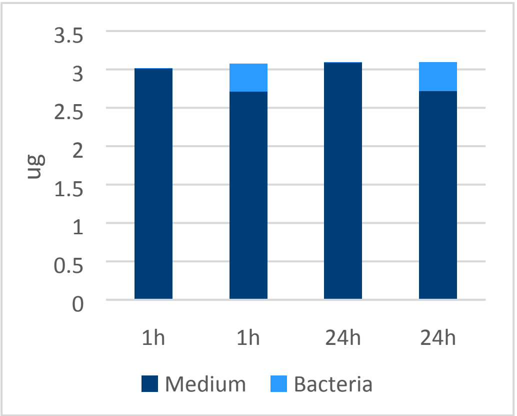

Figure S6: Mass balances for bacteria and medium

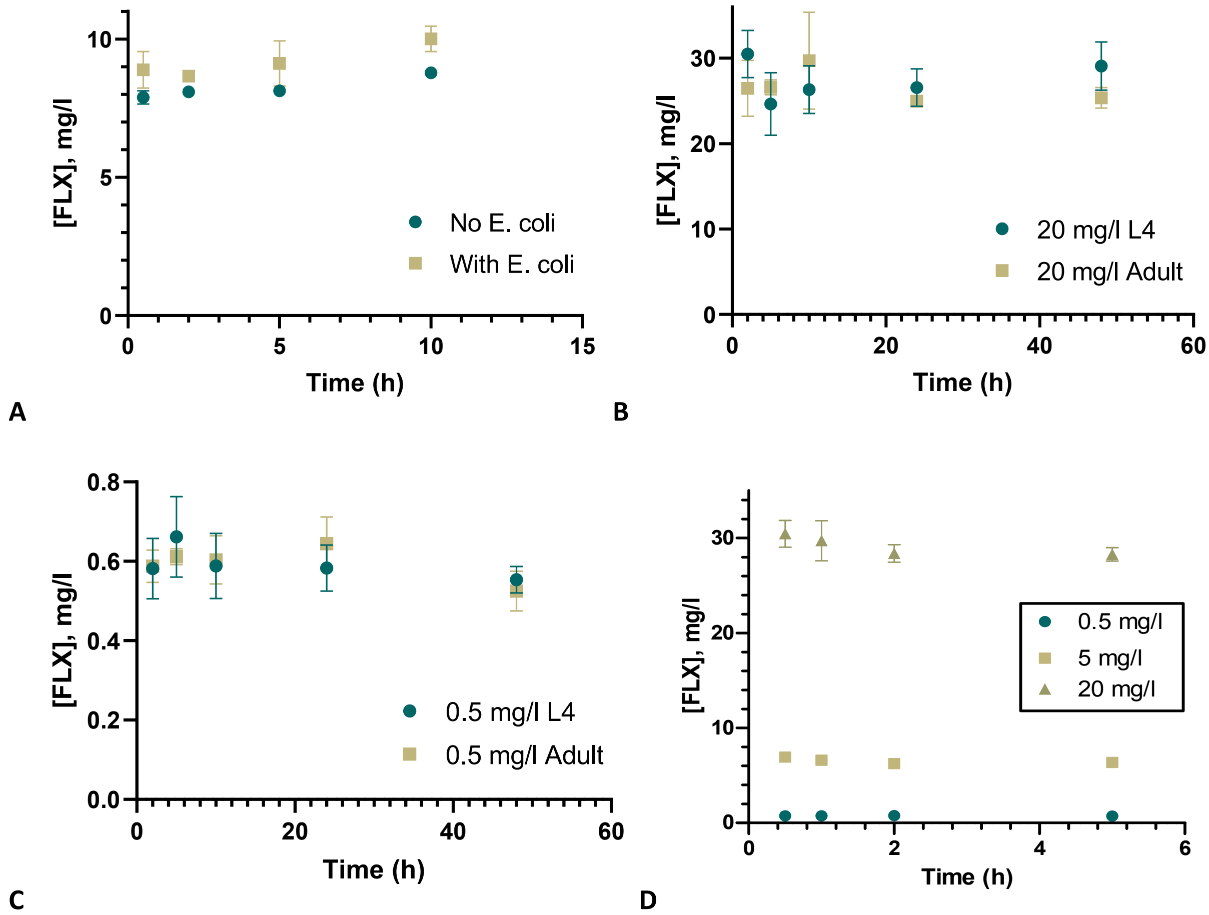

Figure S7: Medium concentrations during experiments: A) experiment III testing the effect of *E. coli*, B) experiment IV for 20 mg/l exposure and C) experiment IV for 0.5 mg/l exposure and D) experiment II for short term exposure without *E. coli*

Table S4: Medium concentrations for experiments

Experiment II: 2h experiment without E. coli		
**Nominal concentration**	**Measured concentration**	**Standard deviation**
**0.5 mg/l**	0.75	0.033
**5 mg/l**	6.53	0.38
**20 mg/l**	29.2	1.53
Experiment III: compare with and without E. coli		
**10 mg/l no bacteria**	8.22	0.38
**10 mg/l with bacteria**	9.17	0.73
Experiment IV: long term (48h) with E. coli		
**0.5 mg/l**	0.59	0.060
**20 mg/l**	27.0	2.88

SI A5: Uptake of norfluoxetine in *C. elegans* compared to fluoxetine

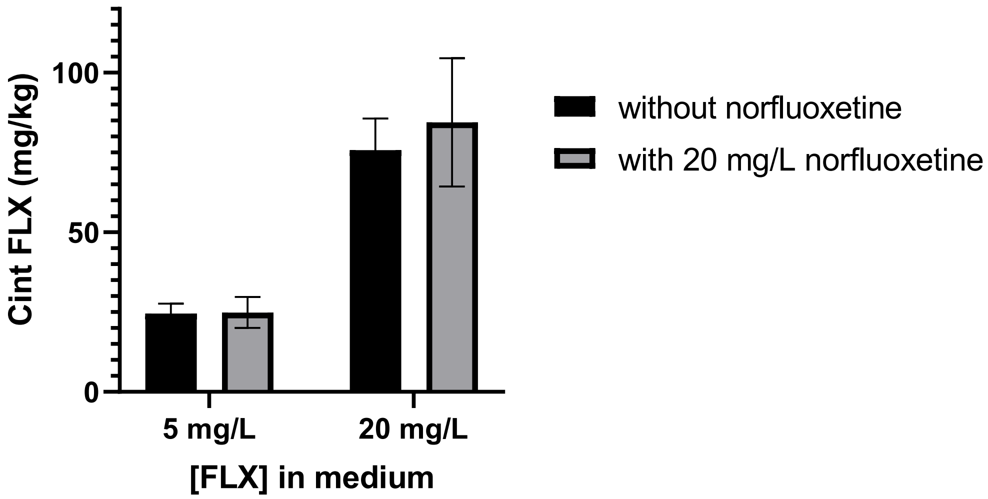

Figure S8: Fluoxetine uptake of *C. elegans* after 5 hour exposure to 5 or 20 mg/L fluoxetine with or without 20 mg/L norfluoxetine

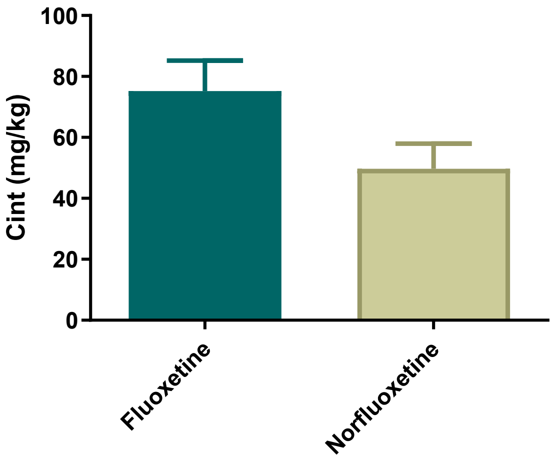

Figure S9: Uptake of 20 mg/L fluoxetine and 20 mg/L norfluoxetine after 5 hours exposure (same exposure as Figure S8)

SI A6: Medium concentrations of norfluoxetine and excretion rates over time

Table S5: Norfluoxetine medium concentrations for experiments III and IV

	Average norflx medium conc (mg/L)				Standard deviation			
	0.5 mg/L	10 mg/L no E coli	10 mg/L	20 mg/L	0.5 mg/L	10 mg/L no E coli	10 mg/L	20 mg/L
**5h**		0.0019	0.0014			0.0012	0.0002	
**10h**	0.0006	0.0040	0.0087	0.0754	0.0003	0.0009	0.0008	0.0317
**24h**	0.0025	0.0140	0.0386	0.3269	0.0011	0.0019	0.0072	0.0321
**48h**	0.0074			0.8398	0.0023			0.0679

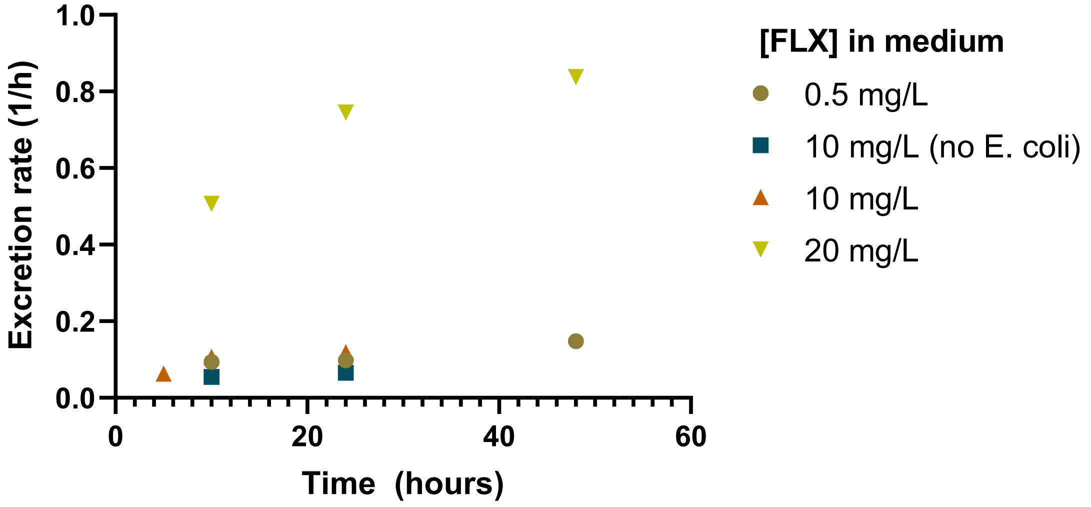

Figure S10: Excretion rates of norfluoxetine, calculated with norfluoxetine medium concentrations (Table S5) and internal norfluoxetine concentrations (see manuscript Figure 4)

SI A7: Changes in cyp35a2 gene expression over time upon fluoxetine exposure

Experimental details:

- Primers cyp35a2
  - Forward: TTCTCCCTTCAAGCATTTAGGA
  - Reverse: ATCGAAAAATTCAGAGGCATGT
- Primers cdc-42
  - Forward: CTGCTGGACAGGAAGATTACG
  - Reverse: CTCGGACATTCTCGAATGAAG
- RT-qPCR details
  - Temperatures:
    - Denaturation - 95°C for 10 seconds
    - Annealing – 60°C for 15 seconds
    - Extension – 72°C for 20 seconds

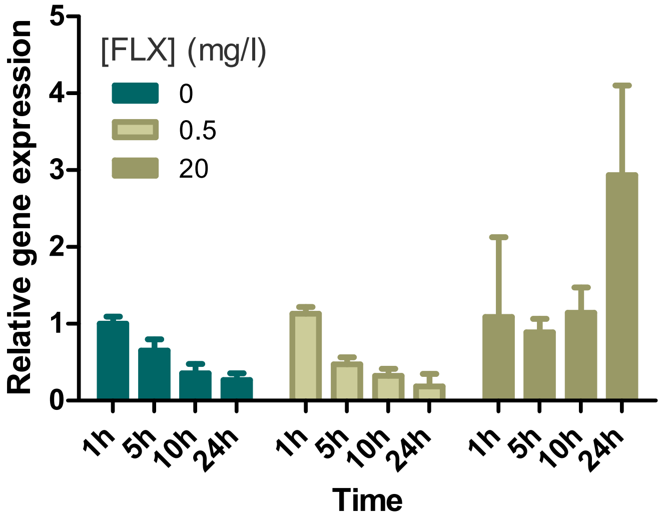

Figure S11: Relative gene expression of the *cup35a2* gene in *C. elegans* compared to housekeeping gene *cdc-42* after exposure to fluoxetine, normalized to 0 mg/l 1h

SI A8: Parameter space plots used to determine confidence intervals around model parameters

Elimination experiment I

Fluoxetine

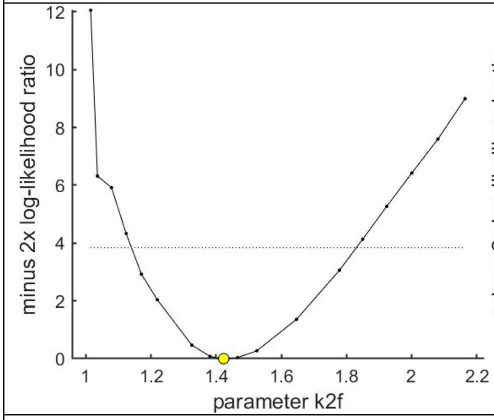

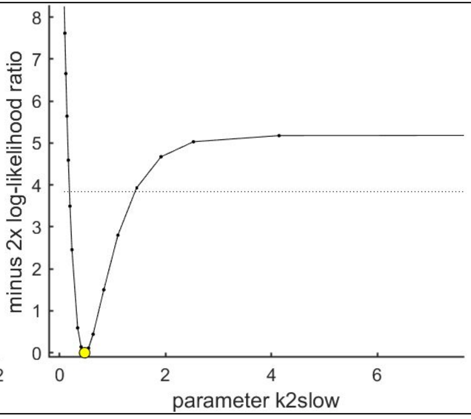

Norfluoxetine

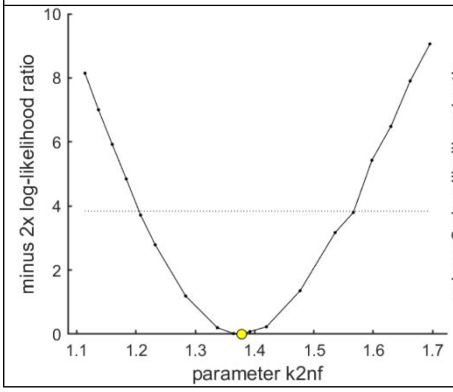

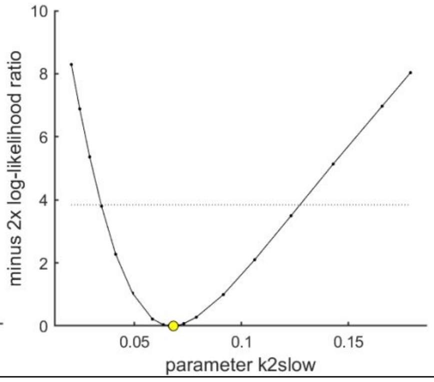

Short uptake (2h) experiment II

0.5 mg/l

Not fixed

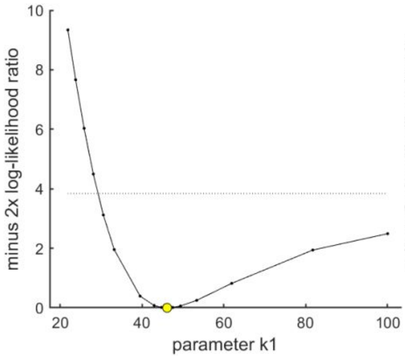

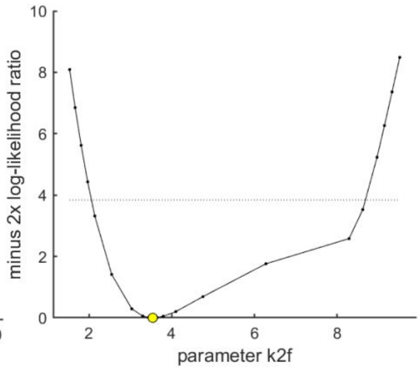

Fixed k2

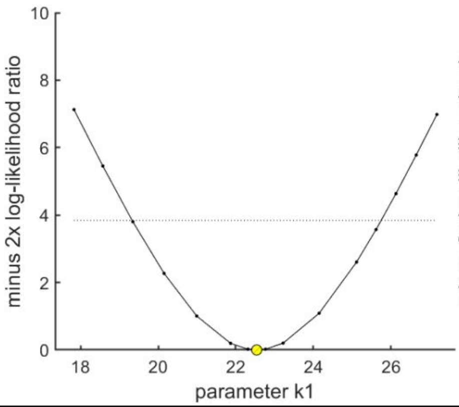

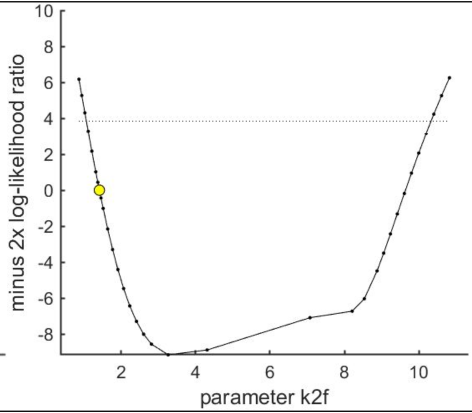

5 mg/l

Not fixed

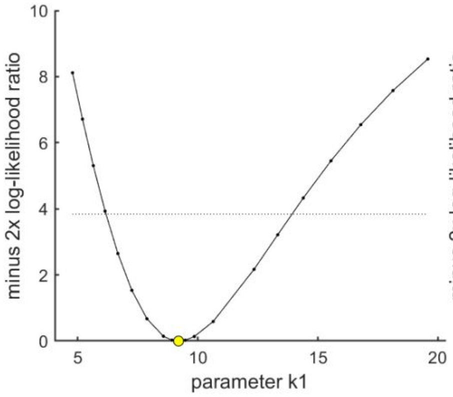

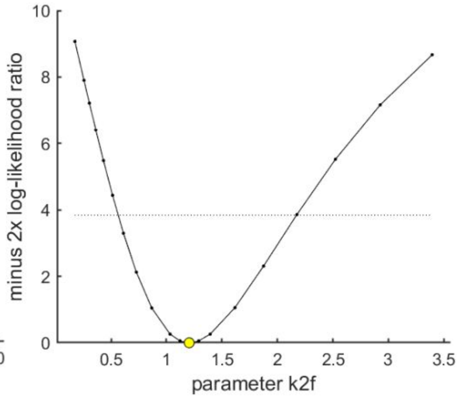

Fixed k2

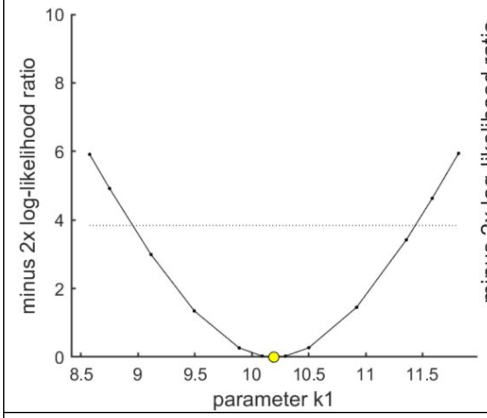

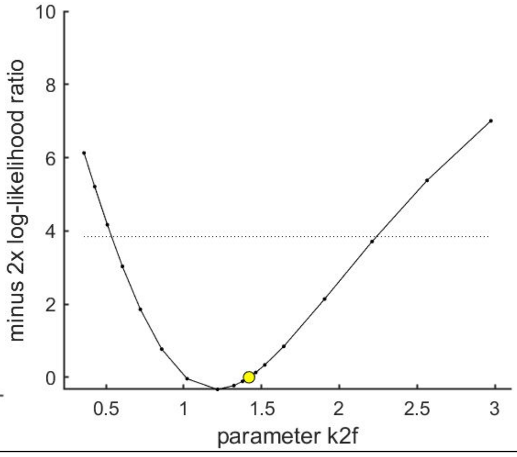

20 mg/l

Not fixed

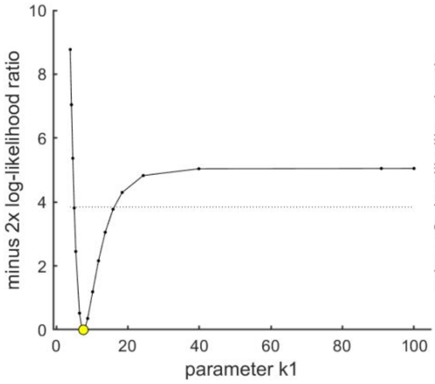

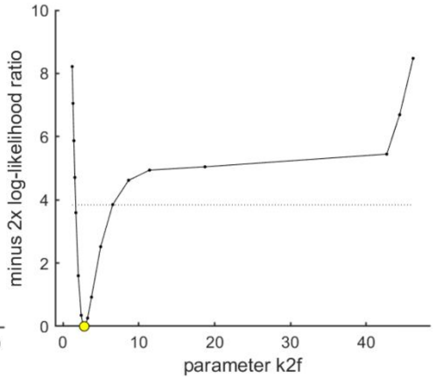

Fixed k2

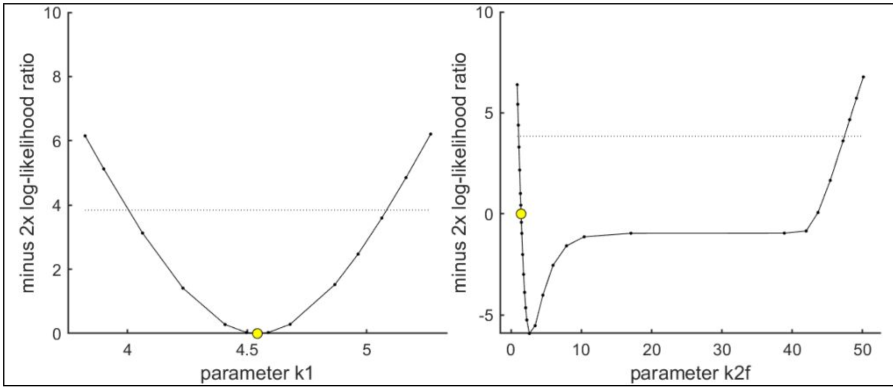

**Experiment III: comparing with and without *E. coli* for 10 mg/l**
**Without E.coli - Equation 1a**
<u>Not fixed</u>

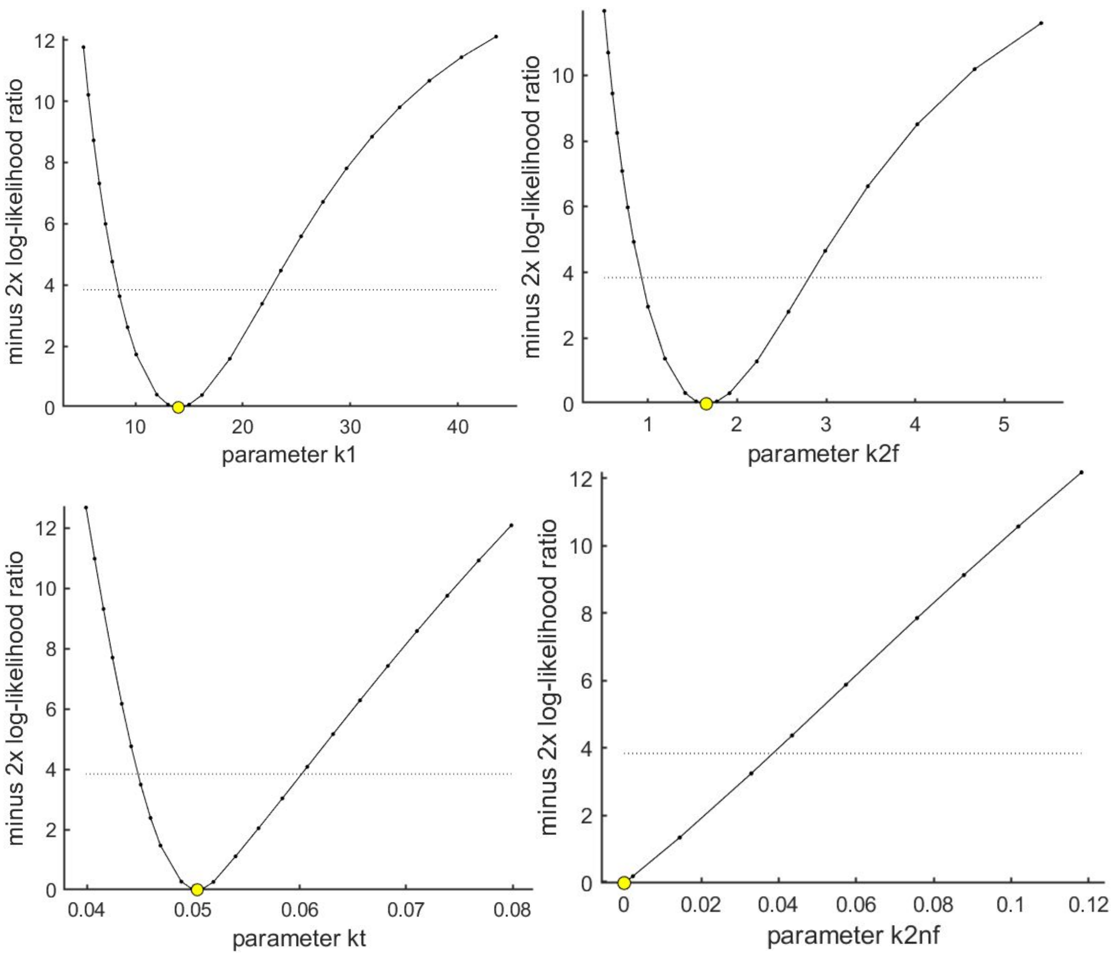

With E.coli - Equation 1a

<u>Not fixed</u>

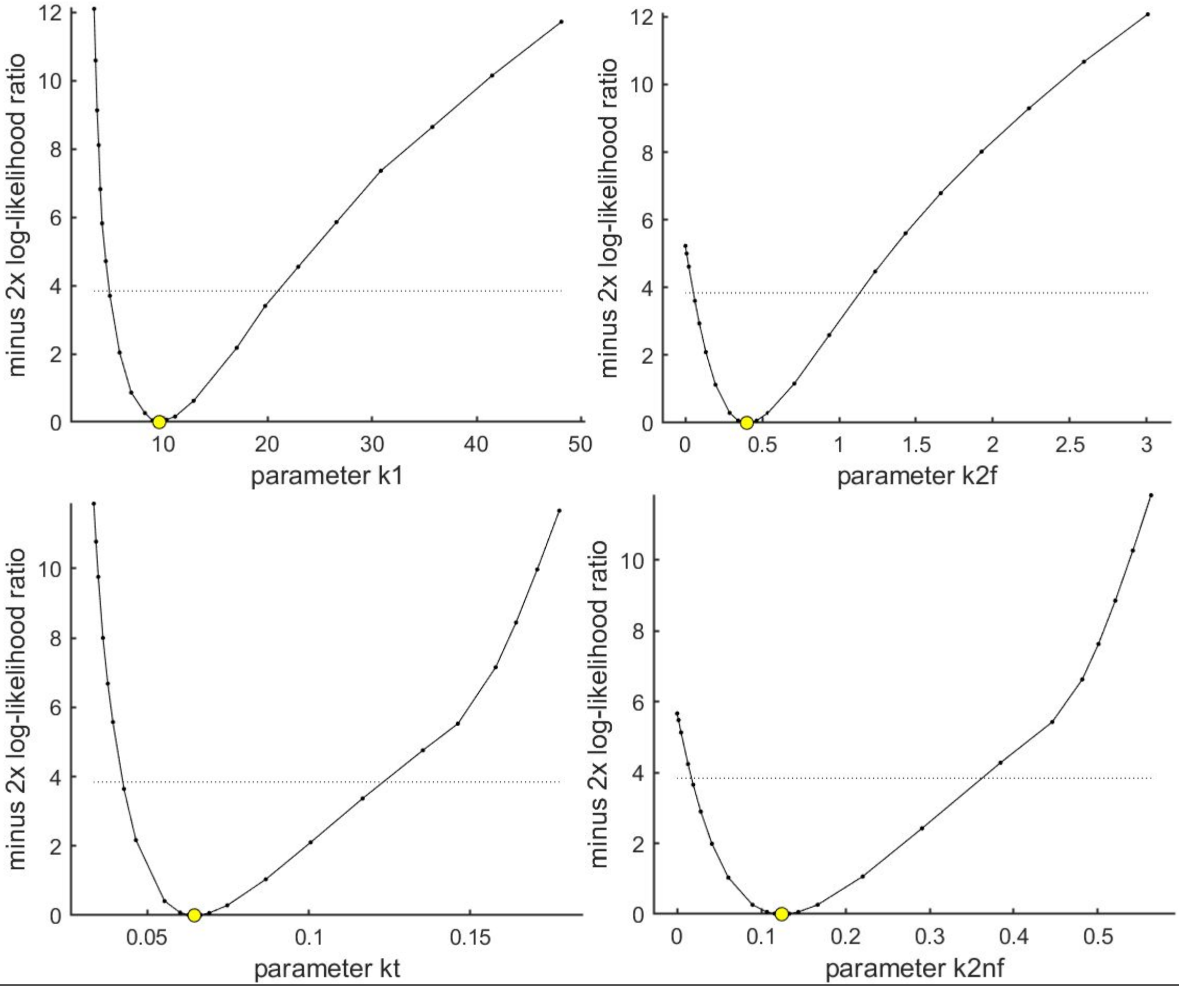

With E. coli – with bacterial ingestion – equation 1b

Experiment IV: Long term (with 0.5 and 20 mg/l)

0.5 mg/l, with equation 1a, k3=ke

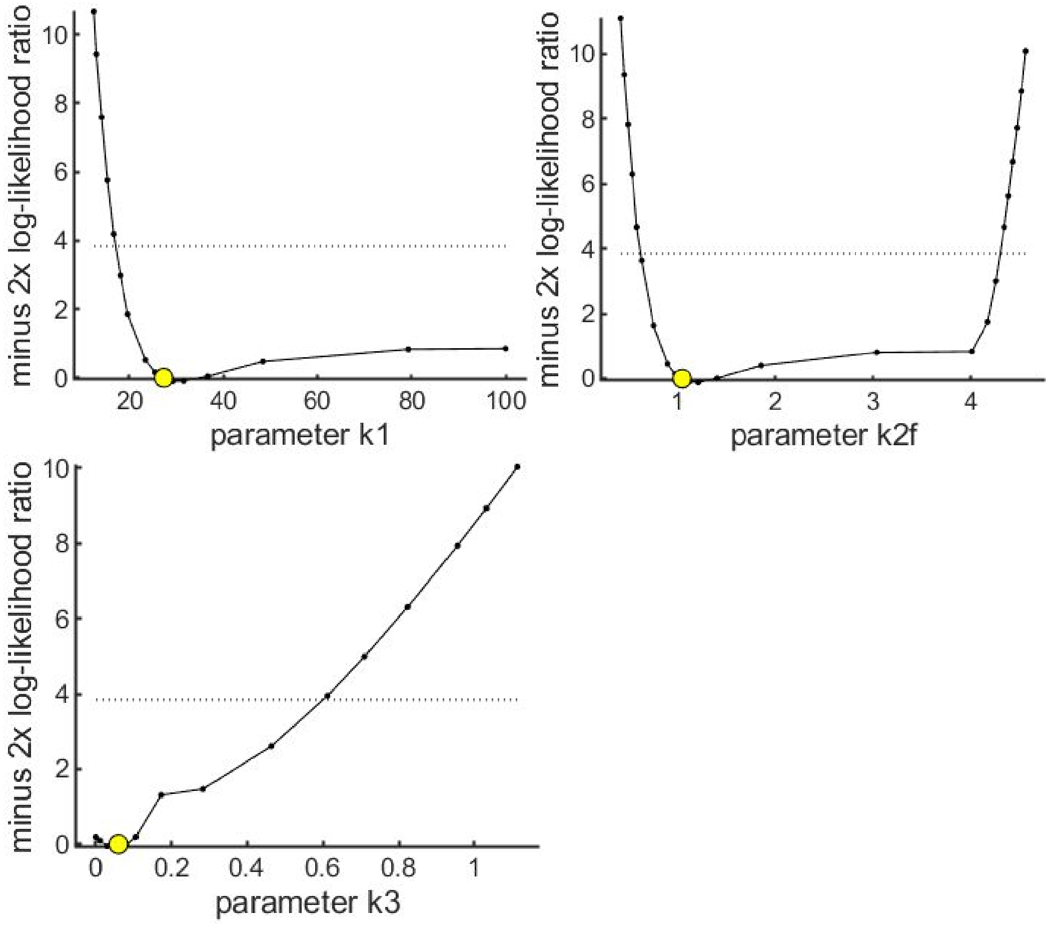

20 mg/l, with equation 1a, k3=ke

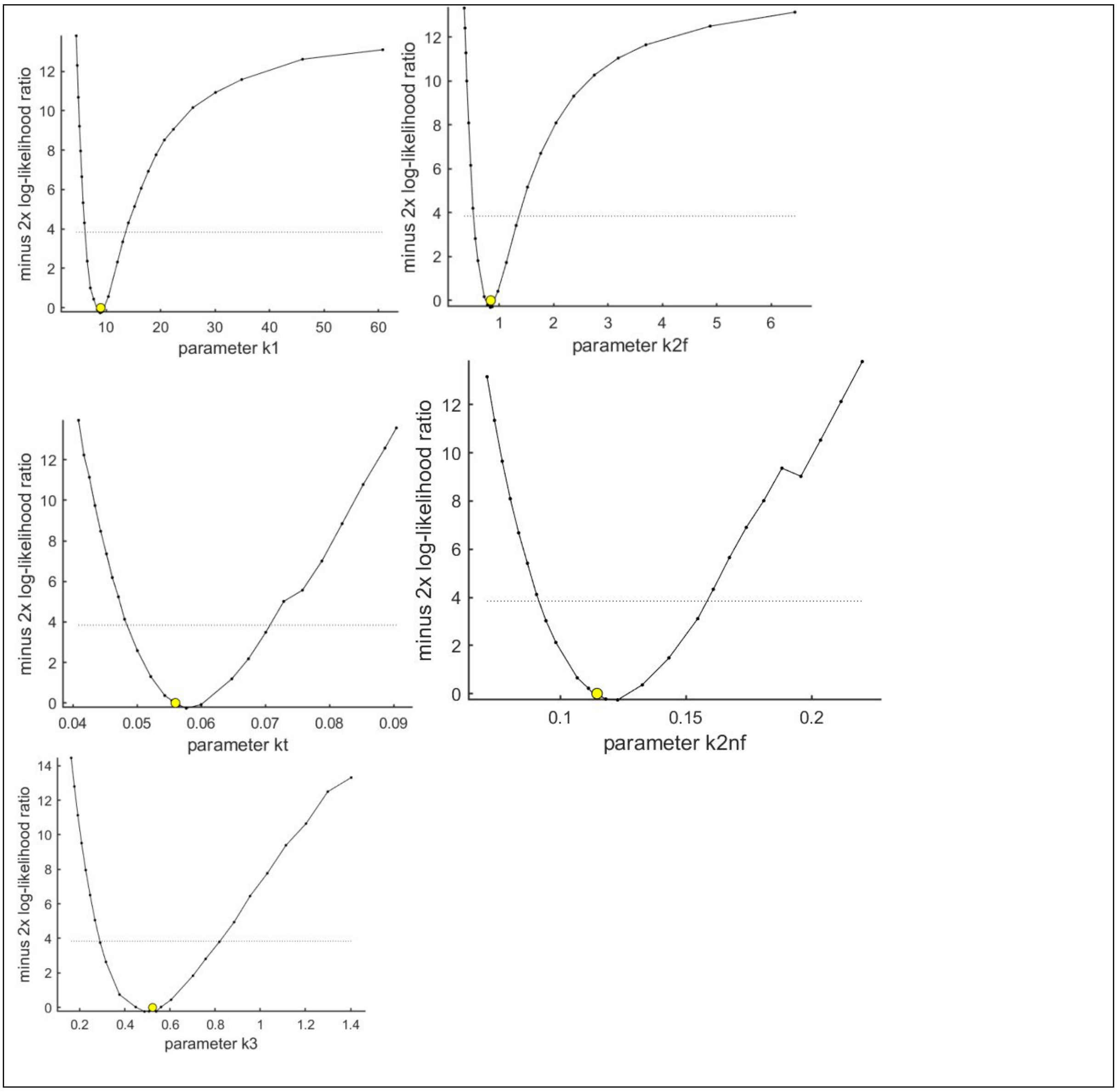

minus 2x log-likelihood ratio
parameter k1
parameter k2f
parameter kt
parameter k2nf
parameter k3

SI A9: MATLAB model scripts based on BYOM modelling package: derivatives

Scripts based on BYOM modelling package version 6.3 (http://www.debtox.info/byom.html). Changes in derivative functions indicated below. Further changes to the script can be made available upon request.

Experiment I: Elimination

Fluoxetine

```
if t < 1
    dCflx = -k2f * Cflx;
else
    dCflx = -k2slow * Cflx;
end
```

Experiment II: Short uptake (2h) for 0.5, 5 and 20 mg/l

```
dCflx = k1 * Cw - k2f * Cflx;
```

Experiment III: comparing with and without *E. coli* for 10 mg/l

10 mg/l without and E.coli - Equation 3a and 5

```
dCflx = k1 * Cw - (k2f + kt) * Cflx;
dCnf = kt * Cflx - k2nf * Cnf;
```

With E. coli – with bacterial ingestion

```
dCflx = k1 * Cw + AE * IR * Cb - (k2f + kt) * Cflx;
dCnf = kt * Cflx - k2nf * Cnf;
```

Experiment IV: Long term (with 0.5 and 20 mg/l)

20 mg/l and 5 mg/l, with km (k3=km)

```
if t < 10
    dCflx = k1 * Cw - (k2f + kt) * Cflx;
else
    dCflx = k1 * Cw - (k2f + kt + k3) * Cflx;
end

dCnf = kt * Cflx - k2nf * Cnf;
```

20 mg/l, with ingestion rate and effect on feeding

```
if t < 10
    dCflx = k1 * Cw + AE * IR * Cb - (k2f + kt) * Cflx;
else
    dCflx = k1 * Cw + EF * AE * IR * Cb - (k2f + kt) * Cflx;
end
dCnf = kt * Cflx - k2nf * Cnf;
```

Parameter/state in script	Parameter in Table S4	Unit
k2f	$k2_{FLX}$	(1/h)
Cflx	$C_{int,FLX}$	(mg flx/kg nem)
k1	k1	$(L/(kg_{nem}*h))$
Cw	C_{med}	(mg flx/L med)
kt	kt	(1/h)
k2nf	$k2_{NF}$	(1/h)
Cnf	$C_{int,NF}$	(mg nf/kg nem)
AE	AE	(0 - 1)
IR	IR	(kg bact/kg nem/hour)
Cb	C_{bact}	(mg flx/kg bact)
k3	ke	(1/h)
EF	EF	(0 - 1)
